# Metabolomic profiling in phenylketonuria: a systematic review of human studies

**DOI:** 10.1007/s11306-026-02416-6

**Published:** 2026-03-07

**Authors:** Arnau Gonzalez-Rodriguez, Mireia Urpi-Sarda, Blanca Barrau-Martinez, Francesc M. Campins-Machado, Hadia Bakkali-Aissaoui, Adriana Pané, Pedro J. Moreno, Emilio Ortega, Judit Garcia-Villoria, Aida Ormazabal, Dolores Garcia-Arenas, Carme Junqué, Gloria Garrabou, Rafael Llorach, the Consortium PKU.cat

**Affiliations:** 1 Departament de Nutrició, Ciències de l’Alimentació i Gastronomia, Facultat de Farmàcia i Ciències de l’Alimentació, Universitat de Barcelona (UB), Campus de l’Alimentació de Torribera, 08921 Santa Coloma de Gramenet, Spain; 2 Institut de Recerca en Nutrició i Seguretat Alimentària (INSA-UB), Universitat de Barcelona (UB), Campus de l’Alimentació de Torribera, 08921 Santa Coloma de Gramenet, Spain; 3 Centro de Investigación Biomédica en Red de Fragilidad y Envejecimiento Saludable (CIBERFES), Instituto de Salud Carlos III (ISCIII), 28029 Madrid, Spain; 4 Endocrinology and Nutrition Department, Adult Inherited Metabolic Disorders Unit (UECMA), Hospital Clínic de Barcelona, 08036 Barcelona, Spain; 5 Centro de Investigación Biomédica en Red de Fisiopatología de la Obesidad y Nutrición (CIBEROBN), Instituto de Salud Carlos III (ISCIII), 28029 Madrid, Spain; 6 Internal Medicine Department, Adult Inherited Metabolic Disorders Unit (UECMA), Hospital Clínic de Barcelona, 08036 Barcelona, Spain; 7 Inherited Metabolic Diseases and Muscle Disorders Research Laboratory, Centre de Recerca Biomèdica CELLEX - Institut d’Investigacions Biomèdiques August Pi i Sunyer (IDIBAPS), Faculty of Medicine and Health Sciences, University of Barcelona, 08036 Barcelona, Spain; 8 Centro de Investigación Biomédica en Red de Enfermedades Raras (CIBERER), Instituto de Salud Carlos III (ISCIII), 28029 Madrid, Spain; 9 Section of Inborn Errors of Metabolism-IBC, Biochemistry and Molecular Genetics Department, Hospital Clínic de Barcelona, IDIBAPS, 08036 Barcelona, Spain; 10 Clinical Biochemistry Department, Sant Joan de Déu Hospital, Institut de Recerca Sant Joan de Déu, Esplugues de Llobregat, 08950 Barcelona, Spain; 11 Inborn Errors of Metabolism Unit, Sant Joan de Déu Hospital, Esplugues de Llobregat, 08950 Barcelona, Spain; 12 Fundació de Recerca Clínic Barcelona-Institut d’Investigacions Biomèdiques August Pi I Sunyer (FRCB-IDIBAPS), Institute of Neurosciences, Department of Medicine, University of Barcelona, Centro de Investigación Biomédica en Red sobre Enfermedades Neurodegenerativas (CIBERNED), 08036 Barcelona, Spain

**Keywords:** Biomarker, Inborn error of metabolism, Metabolite, Metabolomic fingerprint, Pathway analysis, Phenylketonuria

## Abstract

**Background:**

Phenylketonuria (PKU) is a rare metabolic disorder caused by a deficiency in the enzyme phenylalanine hydroxylase, leading to the accumulation of phenylalanine (Phe). Raised Phe levels can result in neurocognitive deficits, intellectual disabilities, and behavioral or psychiatric disorders.

**Aim of review:**

To conduct a systematic review of human studies on metabolites identified through metabolomics in individuals with PKU, compared to healthy controls, and to provide insights into their biological significance.

**Key scientific concepts of review:**

A total of 26 human studies analyzing metabolites in urine and blood met the inclusion criteria. In total, 544 metabolites that differed between patients with PKU and healthy controls were identified through different metabolomic techniques (LC-MS, GC-MS, NMR). Differences were primarily observed in blood samples, which accounted for 95% of the total metabolites, with only 5% detected in urine samples, reflecting the limited use of this body fluid in only five studies. We found 60% of blood metabolites upregulated in patients with PKU, including Phe, Phe-related metabolites, lipids, and other amino acids, while tryptophan and kynurenine, among others, were downregulated (40%). Additionally, 35 metabolites (6% of the total) exhibited inconsistent directions of change (both up- and downregulated), including amino acids, carnitine derivatives, and lipids. These findings may be attributed to clinical factors (dietary adherence, supplementation, and treatment) and methodological differences in blood-derived matrices. Consequently, the high heterogeneity across studies, biological matrices and analytical platforms represents limitations for establishing a unique metabolomic signature. Overall, these results emphasize the metabolic complexity of PKU and highlight the potential of metabolomics to advance disease monitoring and management.

**Supplementary Information:**

The online version contains supplementary material available at 10.1007/s11306-026-02416-6.

## Introduction

**Fig. 1:**
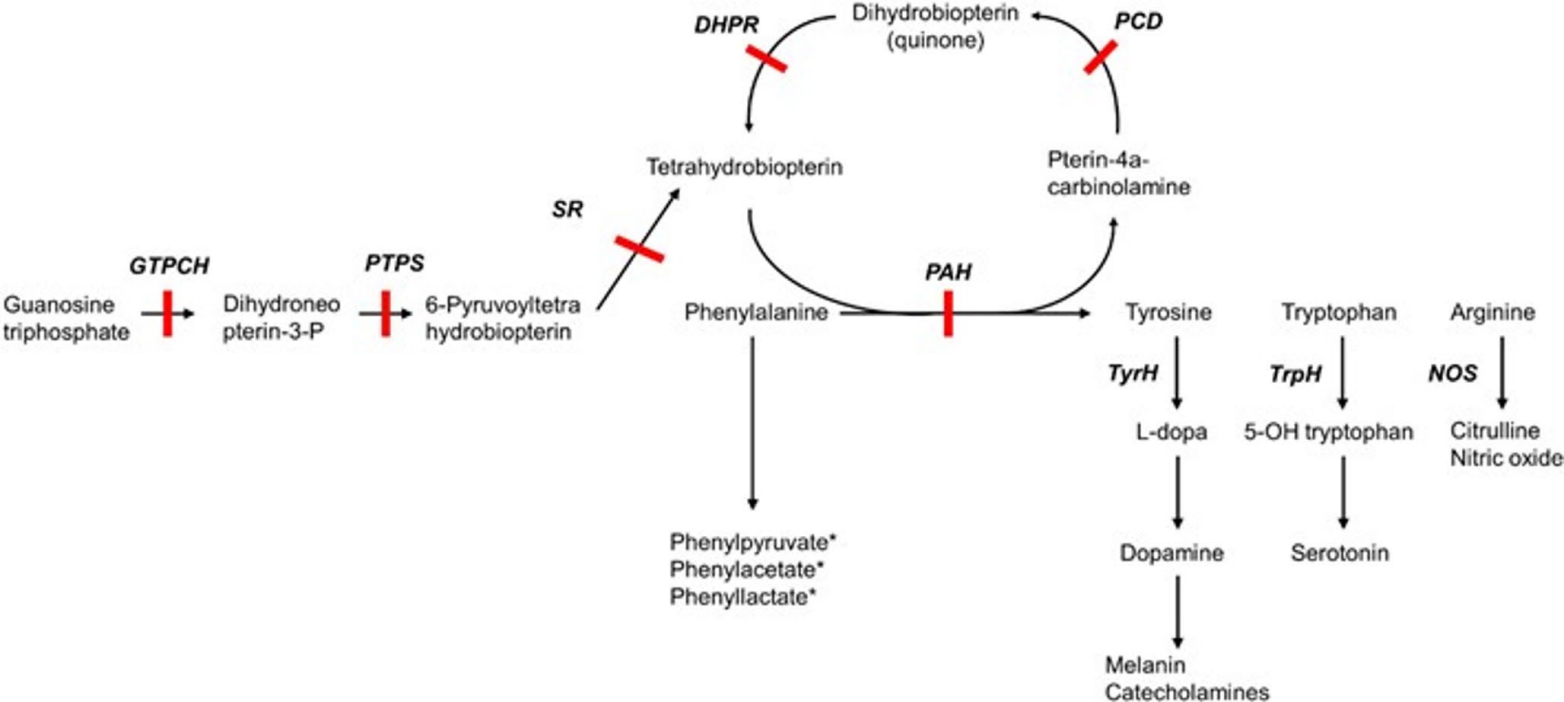
Pathophysiology of Phenylketonuria: Phenylalanine and Tetrahydrobiopterin (BH₄) Metabolism. *Increased systemic concentrations due to high Phe levels. Solid red bars indicate enzyme defects. Adapted from Hollak & Lachmann, 2016. DHPR, dihydropteridine reductase; GTPCH, guanosine triphosphate cyclohydrolase; NOS, nitric oxide synthase; PAH, phenylalanine hydroxylase; PCD, pterin-4a-carbinolamine dehydratase; PTPS, pyruvoyl-tetrahydrobiopterin synthase; SR, sepiapterin reductase; TrpH, tryptophan hydroxylase; TyrH, tyrosine hydroxylase

Phenylketonuria (PKU; OMIM #261600) is a rare inborn error of metabolism (IEM) caused by pathogenetic variants in the gene encoding the enzyme phenylalanine hydroxylase (PAH) (van Wegberg et al., 2017, 2025). Despite being considered rare, PKU is one of the most frequent aminoacidopathies, with an estimated 0.45 million affected individuals and a global prevalence of approximately 1:23,930 live births (Hillert et al., 2020). These mutations lead to PAH deficiency. PAH converts phenylalanine (Phe) to tyrosine (Tyr) in a reaction that requires its cofactor, tetrahydrobiopterin (BH4), as well as oxygen and iron (Fig. 1). PAH deficiency leads to substantially increased blood Phe concentrations and toxic levels in the brain of patients with PKU. As a result, the risk of Tyr deficiency is elevated, potentially causing neurotransmitter dysfunction (Blau et al., 2010; van Spronsen et al., 2021). However, certain PAH mutations only partly inhibit PAH activity, leading to lower rises in Phe concentrations and resulting in mild hyperphenylalaninemia (HPA) or mild PKU (Blau et al., 2010; van Wegberg et al., 2017). Consequently, untreated patients develop neurocognitive deficits (Rausell et al., 2019), as well as psychiatric, behavioral and movement disorders that become more evident as the child grows (Blau et al., 2010; van Spronsen et al., 2021). Therefore, early diagnosis of PKU through newborn screening (NBS) programs is critical to ensure proper metabolic control and optimal neurocognitive functioning (Rausell et al., 2019; van Wegberg et al., 2017, 2025). PKU management involves chronic dietary changes and, for BH4-responsive patients, BH4 therapy to control Phe levels (Blau et al., 2010; van Wegberg et al., 2017). To manage their condition, patients often rely on specially formulated protein-free substitutes, special foods, and restrict foods high in natural protein (Blau et al., 2010; MacDonald et al., 2020; Rondanelli et al., 2023).

NBS worldwide for PKU has been a cost-effective diagnostic method, enabling early treatment and leading to the best possible outcomes for patients (van Spronsen et al., 2021; van Wegberg et al., 2017, 2025). NBS usually consists of the biochemical analysis of a dried blood spot (DBS) obtained in the first week of life (van Spronsen et al., 2021). The diagnosis of PKU is conducted through targeted metabolomics, measuring specific metabolites (amino acid profile and organic acids) using various chromatographic and mass spectrometry (MS) systems. However, untargeted metabolomics using high resolution mass spectrometry (HRMS) is gradually emerging in the diagnosis of IEMs, including PKU (Mordaunt et al., 2020), as it can analyze thousands of metabolites in a single test. In this context, nuclear magnetic resonance (NMR) spectroscopy or HRMS can detect, identify and quantify metabolites (Ulaszewska et al., 2019) to assess the affected metabolic pathways, providing insight into the relationships between metabolites and health status.

Despite NBS and early treatment, some patients are less responsive to the treatment. Integrating targeted nutritional strategies with metabolomic profiling could facilitate the identification of individualized biomarkers, leading to more effective dietary interventions and improved clinical outcomes for this population. Consequently, metabolic profiling of patients with PKU may have the potential to personalize medicine and contribute to enhance their quality of life (Ulaszewska et al., 2019).

The aim of this work was to conduct a systematic review of human studies on metabolites identified through metabolomics in individuals with PKU, compared to healthy controls, and to provide insights into their biological significance.

## Materials and methods

### Search strategy and study selection

The systematic literature review was conducted by searching PubMed^®^, Web of Science™, and Scopus^®^ databases, considering publications available up to May 2023. The search syntax employed for each database is detailed in Supplementary Table 1.

The inclusion criteria for studies in this systematic review were defined as follows: (a) the provision of biological insights into PKU through the application of metabolomics techniques; (b) human subjects; (c) use of metabolomics approach for the analysis of blood and urine human samples; (d) comparison with healthy controls; (e) the full-text article being written in English; and (f) the full-text being available. Studies conducted solely for newborn screening purposes that measured only Phe or the Phe/Tyr ratio and review articles were excluded. Notably, there were no restrictions imposed on subject characteristics such as age, sex, weight, or other health conditions. Additional articles were selected by reviewing the bibliographies of the included studies.

### Data extraction

Data were extracted from each included study, comprising reference details, participant and control subject numbers, used metabolomic technique, analyzed metabolites, and their respective up- or downregulation in patients with PKU.

### Quality assessment

The methodological quality of the included studies was assessed using the QUADOMICS instrument (Lumbreras et al., 2008), which is an adaptation of QUADAS (Quality Assessment of Diagnostic Accuracy Studies) (Whiting et al., 2003). This instrument employs a 16-item scale that evaluates various aspects such as inclusion and exclusion criteria, sample characteristics, preanalytical conditions of the sample, clinical and physiological characteristics of the patients, confirmation of the diagnosis, occurrence of uninterpretable test results, and the presence or avoidance of over-fitting, among others (Supplementary Table 2). The QUADOMICS tool was applied following a systematic review (Carrard et al., 2022). Each item was assigned a score of 1 if clearly described, 0.5 points if unclear, and 0 points if not properly explained. A score of 70% was selected as a threshold value to include the studies in the systematic review (Hou et al., 2023).

### Pathway analysis of metabolites

MetaboAnalyst 6.0 (Pang et al., 2024) Pathway Analysis module was used to perform a complementary analysis of the metabolic pathways affected from the list of metabolites. The Kyoto Encyclopedia of Genes and Genomes (KEGG) (Kanehisa et al., 2016) pathway library for *Homo sapiens* was selected in the MetaboAnalyst software to analyze the different pathways affected. Overrepresentation analysis was performed using the Hypergeometric test. Relative-betweenness centrality was selected as a topology measure. A total of 235 metabolites in blood had a metabolic pathway assigned by the software. Metabolites up- and downregulated from blood were analyzed separately. Pathways were considered statistically significant if the False Discovery Rate (FDR) was less than 0.05.

## Results

### Selection of studies

**Fig. 2:**
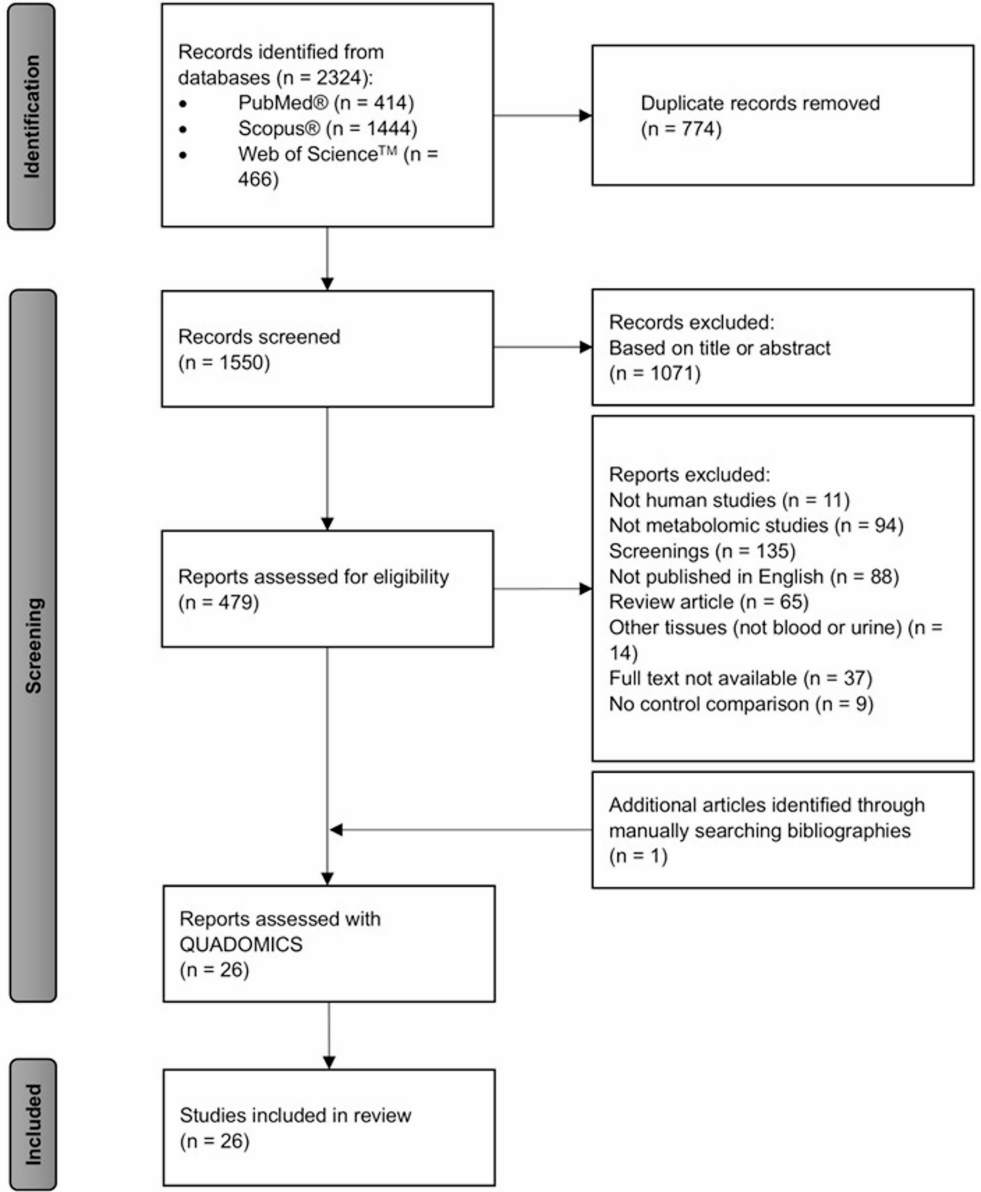
Flowchart of the systematic review adapted from PRISMA 2020 model (Page et al., 2021). PKU, phenylketonuria; PRISMA, Preferred Reporting Items for Systematic Reviews and Meta-Analyses

In total, 2324 articles were identified from the three previously mentioned databases, and one additional study was included through manual search. After removing duplicates, 1550 articles remained, and their titles and abstracts were screened for eligibility. Twenty-five studies (Andrade et al., 2017; Blasco et al., 2017; Bonte et al., 2019; Boulet et al., 2020; Cannet et al., 2020; Coene et al., 2018; Douglas et al., 2013; Drzymała-Czyż et al., 2018; Guerra et al., 2021; Haijes et al., 2019; Hampe et al., 2017; Hoegen et al., 2022; Jacob et al., 2018; Kong & Hernandez-Ferrer, 2019; Liang et al., 2020; Miller et al., 2015; Moritz et al., 2023; Mütze et al., 2012; Pan et al., 2007; Schoen & Singh, 2022; Schulpis et al., 2002; Václavík et al., 2018; Wan et al., 2022; Weigel et al., 2008; Xiong et al., 2015) were identified from the mentioned databases, and an additional article (Stroup et al., 2018) was incorporated after searching through the reference lists. Finally, 26 studies were included in the systematic review after quality assessment (Fig. 2).

### Characteristics of the selected studies

Table 1 provides an overview of the 26 studies included in this review, summarizing participant characteristics, metabolomics techniques applied, analyzed biosample and the main differential metabolites reported when comparing PKU individuals with controls.

**Table 1:** Characteristics of the selected studies

Author	Participants characteristics	Metabolomic technique	Sample (collection time)	[↑] Metabolites in PKU vs. control	[↓] Metabolites in PKU vs. control
Andrade et al. (2017)	42 patients with PKU (19 F-23 M; P_3_: 2 year; P_97_: 36 year) and 40 age- and sex- matched healthy controls. 33 patients treated with Phe-restricted diet and 9 patients with diet and KUVAN.	RP-HPLC-QQQ-MS (Targeted analysis)	Plasma (Fasting)	Phenylalanine Cysteine Glycine Ornithine	Asymmetric dimethylarginine Symmetric dimethylarginine Arginine
Blasco et al. (2017)	10 adults with PKU (6 F-4 M; 18–39 year) and 10 age- and sex- matched healthy controls. No Phe-restricted diet at inclusion.	AminoTac 500 analyzer based on the ion-exchange chromatography with a post-column derivatization with ninhydrin and colorimetric detection (Targeted analysis)	Plasma (Fasting)	Phenylalanine	Tyrosine Isoleucine Glutamine Arginine Proline Alpha aminobutyric acid Threonine Methionine Alanine
		AminoTac 500 analyzer based on the ion-exchange chromatography with a post-column derivatization with ninhydrin and colorimetric detection; organic acids with GC-MS and metabolomics profile with NMR (Targeted analysis)	Urine (Fasting)	3-Hydroxymethylglutaric acid Isovalerylglycine 2-Methyl succinic acid Fumaric acid	Succinic acid Methylmalonic acid Glyceric acid 3-hydroxyisovaleric acid Pyruvic acid
Bonte et al. (2019)	3 individuals with PKU (age and gender not reported) and age- and sex-matched control individuals. Diet treatment not reported in the study.	RP-UPLC-Orbitrap-MS (Untargeted analysis)	Plasma (Unspecified)	Gamma-glutamylphenylalanine N-acetylphenylalanine N-lactoylphenylalanine Phenylacetic acid Phenylalanine Phenylalanylphenylalanine Phenyllactic acid Phenylacetylglutamine	Not reported
Boulet et al. (2020)	151 adults with PKU (97 F-54 M; 18–45 year; mean: 26.8 year) and 30 healthy controls (20 F-10 M; mean: 39 year) 85 patients were under low-Phe diet (69 with AA-MF)	RP-HPLC-MS/MS (Targeted analysis)	Plasma (Fasting)	Phenylalanine Kynurenic acid	Kynurenine 3-hydroxykynurenine
Cannet et al. (2020)	22 adults with PKU (16 F-6 M; 30–54 year) and 14 healthy controls (8 F-6 M; 30–45 year) 15 subjects had good adherence to their Phe-restricted diet.	NMR (Targeted analysis)	Plasma (Fasting)	Phenylalanine Citric acid Glutamic acid	Glutamine Tyrosine Creatinine
Coene et al. (2018)	10 individuals with PKU; age and gender not reported) and age- and sex-matched control individuals. Patients were on a Phe-restricted diet.	RP-UPLC-QTOF-MS (Targeted and untargeted analysis)	Plasma (Unspecified)	Phenylalanine Gamma-glutamylphenylalanine Phenyllactic acid N-(1-deoxy-1-fructosyl)phenylalanine N-acetylphenylalanine Phe-hexose Glutamyl-glutamyl-phenylalanine	Not reported
Douglas et al. (2013)	58 participants with PKU (24 F-34 M; 4–49 year; mean: 17 year) and 13 healthy controls (6 F-7 M; 7–37 year; mean: 20 year) at baseline. Participants were on a Phe-restricted diet.	Biochrom 30 HPLC Amino Acid Analyzer (Targeted analysis)	Plasma (Fasting)	Phenylalanine	Not reported
		RP-HPLC-ED (Targeted analysis)	Urine (Fasting)	Not reported	Dopamine Homovanilic acid 3-methoxytyramine 5-hydroxyindoleacetic acid 5-hydroxytryptamine
Drzymała-Czyż et al. (2018)	40 patients with PKU (20 F-20 M; 11–35 year) and 40 healthy subjects (20 F-20 M; 18–33 year). Patients were adhered to a Phe-restricted diet.	GC-MS (Targeted analysis)	Serum (Fasting)	Stearic acid γ-linolenic acid α-linolenic acid	Mead acid Linoleic acid Eicosadienoic acid Arachidonic acid Docosatetraenoic acid Docosapentaenoic acid Docosahexaenoic acid
Guerra et al. (2021)^a^	15 children with PKU (7 F-8 M; 3–17 year) vs. 12 healthy controls (4 F-8 M; 7–16 year) Patients were following a lifelong Phe-restricted diet. 13 patients had AA mixtures prescribed and 6 patients were PUFA supplemented.	High resolution HILIC-MS/MS and GC-MS (Targeted and untargeted analysis)	Plasma (Fasting)	Myristic acid Stearic acid Oleic acid (18:1n-9) 18:1 Eicosapentaenoic acid Docosahexaenoic acid 13 Phosphatidylcholine (38:7, 44:4, 40:7, 40:5, 40:4, 42:4, 38:5, 44:12, 44:11, 40:8, 42:7, 42:9, 40:9) 5 Sphingomyelin (d36:2, d36:3, d38:3, d34:2, d34:1) 1 Lysophosphatidylcholine (14:0) 2 Phosphatidylinositol (38:4, 36:4)	Sphingomyelin (d32:2) 3 Phosphatidylserine (38:4, 40:6, 38:6)
Haijes et al. (2019)	6 individuals with PKU (age and gender not reported) and control individuals. Diet treatment not reported in the study.	Direct Infusion – High-Resolution Mass Spectrometry (Untargeted analysis)	Dried blood spot (Unspecified)	Phenylalanine N-acetylphenylalanine Hydroxyphenylacetic acid isomer	Tyrosine
Hampe et al. (2017)	6 children with PKU (age and gender not reported) and age-matched controls. Diet treatment not reported in the study.	GC-MS (Targeted analysis)	Urine (Unspecified)	Phenylpyruvic acid Phenylacetic acid Phenyllactic acid 2-Hydroxyphenylacetic acid Phenylalanine Mandelic acid 4-Hydroxyphenyllactic acid	Not reported
Hoegen et al., (2022)	8 individuals with PKU (age and gender not reported) and age- and sex-matched control individuals. Patients were on a Phe-restricted diet.	RP-UPLC-QTOF-MS (Untargeted analysis)	Plasma (Unspecified)	Phenylalanine-acetylphenylalanine *Trans*-cinnamic acid Phenylacetic acid 1-Phenyl-1,2-propanedione	Not reported
Jacob et al. (2018)	15 patients with PKU (8 F-7 M; mean: 13.9 year) and 20 healthy adult controls. Diet treatment not reported in the study.	RP/UPLC-MS/MS (Targeted analysis)	Dried blood spot (Unspecified)	Phenylalanine Carnitine Hydroxyproline Succinate Glutathione Acetylcarnitine Guanosine Uridine-5’-monophosphate Adenosine 3’,5’-monophosphateInosine 3-Phosphoglyceric acid Glutamine Arginine D-Pantothenic acid Methylmalonate Creatinine Phosphoenolpyruvic acid Betaine Fructose 1,6-Bisphosphate Glucose 6-phosphate	Lactate Valine Taurine Niacinamide Guanosine monophosphate Glycine Ethanolamine
Kong and Hernandez-Ferrer (2019)	1 adult with PKU (40 year M) and healthy controls. Patient was following a Phe-restricted diet.	RP/UPLC-MS/MS HILIC/UPLC-MS/MS (Untargeted analysis)	Plasma (Unspecified)	Phenylalanine Phenyllactate Phenylpyruvate 4-hydroxyphenylacetate 2-hydroxyphenylacetate Gamma-glutamylphenylalanine myristoyl-linoleoyl-glycerol (14:0/18:2)	Alpha-ketoglutaramate
Liang et al. (2020)	116 newborns with HPA (52 F-64 M; mean: 5 d) vs. 150 age-matched healthy newborns. Newborns with HPA data before treatment. 90% newborns 120–600 Phe µmol/L	Direct injection LC-MS/MS (Targeted analysis) .	Dried blood spot (Fasting)	Phenylalanine Citrulline Valine Ornithine	Tyrosine Glutamine Threonine
	161 patients with HPA (66 F-95 M; 1 m-5 year; mean: 103 d) vs. 200 age-matched healthy controls. Patients with HPA data before treatment. 54% patients > 600 Phe µmol/L			Phenylalanine Valine Histidine Serine	Tyrosine Threonine Alanine Aspartic acid Glutamic acid Methionine Arginine Glycine Ornithine Glutamine
Miller et al. (2015)	8 patients with PKU (age and gender not reported) and control individuals. Most patients were on a Phe-restricted diet.	RP-UPLC-Orbitrap-MS (Untargeted analysis)	Plasma (Unspecified)	Phenylalanine Phenyllactic acid Gamma-glutamylphenylalanine Phenylpyruvic acid N-acetylphenylalanine	Not reported
Moritz et al. (2023)	28 patients with PKU (15 F-13 M; 2–49 year; 1 with HPA) and 32 healthy controls (23 F-9 M; 19–60 year). 26/28 patients used medical foods, and 2/28 received tyrosine supplementation. 57% had bad metabolic control according to longitudinal DBS Phe values.	RP-LC-MS/MS (Targeted analysis)	Plasma (Unspecified)	Phenylalanine Tyrosine Glutamic acid Aspartate Serine Taurine Hypotaurine Alpha-ketoglutarate Methionine sulfoxide	Citric acid 2-Methylcitric acid Glutathione Homocysteine
Mütze et al. (2012)	12 children with PKU (6 F-6 M; 5–14 year) and 8 healthy controls (5 F-3 M; 5–17 year). Patients with PKU followed their usual diet treatment. Patients were only eligible if they were under good metabolic control.	Flow injection analysis-MS/MS (Targeted analysis)	Dried blood spot (Fasting)	Not reported	Carnitine Acetylcarnitine Propionylcarnitine Hydroxybutyrylcarnitine Isovalerylcarnitine 2-Hydroxyisovalerylcarnitine Hexanoylcarnitine Octanoylcarnitine Decenoylcarnitine Glutarylcarnitine Tetradecenoylcarnitine Hexadecenoylcarnitine Octadecanoylcarnitine 3-Hydroxyoct adecanoylcarnitine Octadecenoylcarnitine Trans, trans-9,12-octadecadienoic acid
		GC with flame ionization and aa by ion exchange chromatography and ninhydrin derivatisation (Targeted analysis)	Plasma (Fasting)	γ-linolenic acid Phenylalanine	Hydroxyproline Asparagine
Pan et al. (2007)	1 adolescent or adult with PKU. Phe-restricted diet since child diagnosis.	NMR and desorption electrospray ionization coupled to mass spectrometry (Targeted analysis)	Urine (Unspecified)	Phenylalanine Citrate Alanine 2-Hydroxyphenylacetic acid	Not reported
Schoen and Singh (2022)^a^	28 patients with PKU (28 F; 15 adults (median: 22 year) + 13 pediatric (median 15 year)) and 28 age-, sex- and self-reported race- matched controls. 15 participants received adjunct pharmacotherapies (10 still consuming AA-MF or GMP-MF); 13 participants only had dietary treatment; 18 participants had Phe concentrations exceeding the desired range.	RP/UPLC-MS/MS and HILIC/UPLC-MS/MS (Untargeted analysis)	Plasma (Fasting)	Phenylalanine N-formylphenylalanine Gamma-glutamylphenylalanine N-acetylphenylalanine Phenyllactate Phenylpyruvate Cysteinylglycine Sarcosine Cysteine-s-sulfate Iminodiacetate 1-carboxyethylphenylalanine 3-formylindole Oleoylethanolamide Linolenoylcarnitine 2-hydroxyphenylacetate	Perfluorooctanesulfonic acid 1-stearoyl-GPI Leucine Oleoylcholine Stearoylcholine Arachidonylcholine Glycerophosphorylcholine Palmitoylcholine Alpha-Linoleoylcholine Pyrraline
Schulpis et al. (2002)	12 children with PKU (mean: 6.8 year) and 23 age-matched healthy controls. Patients were adhered to a Phe-restricted diet.	Amino acid analyzer (Biotronic LC 5001), RP-HPLC-ED and cationic exchange-HPLC (Targeted analysis)	Plasma (Fasting)	Phenylalanine	Not reported
	11 children with PKU (mean: 7.2 year) and 23 age-matched healthy controls. Patients were off-diet.			Phenylalanine	Tyrosine Tryptophan Dopamine Noradrenaline 5-hydroxytryptamine Adrenaline
Stroup et al. (2018)	10 patients with PKU (6 F-4 M; 8 adults and 2 adolescents) vs. 15 age- and sex-matched control participants. Patients followed their usual low-Phe diet + AA-MF. Only 1 participant used sapropterin dihydrochloride.	RP/UPLC-MS/MS and HILIC/UPLC-MS/MS (Untargeted analysis)	Plasma (Fasting)	Phenylalanine Stearidonic acid 13-hydroxyoctadecadienoic acid 9-hydroxyoctadecadienoic acid Phenylacetate Linolenoylcarnitine	Deoxycarnitine Stearoylcarnitine Arachidoylcarnitine Lignoceroylcarnitine Margaroylcarnitine Cerotoylcarnitine
	Same patients with PKU followed their usual low-Phe diet + GMP-MF with a 3-wk washout period Only 1 participant used sapropterin dihydrochloride.			Phenylalanine Stearidonic acid Phenylacetate	Deoxycarnitine Stearoylcarnitine Arachidoylcarnitine Lignoceroylcarnitine Margaroylcarnitine Cerotoylcarnitine
Václavík et al. (2018)	7 patients with PKU (age and gender not reported) and controls. Patients were on a Phe-restricted diet.	LC-Orbitrap-MS and RP-HPLC-QTOF-MS (Targeted and untargeted analysis)	Plasma (Unspecified)	Phenylalanine Gamma-glutamylphenylalanine Phe-hexose Glutamyl-glutamyl-phenylalanine Phenylalanylphenylalanine N-lactoylphenylalanine	Not reported
Wan et al. (2022)	45 neonates with PKU (17 F-28 M;0-4wk) vs. 45 sex-matched healthy neonates. Neonates data before treatment.	HPLC-MS/MS (Targeted analysis)	Dried blood spot (Fasting)	Phenylalanine Arginine Citrulline Valine Methionine	Tyrosine Proline
	27 patients with PKU (9 F-18 M;1–4 year) vs. 27 age- and sex-matched normal subjects. Patients were on a Phe-restricted diet.			Phenylalanine Citrulline Valine	Not reported
Weigel et al. (2008)	30 patients with PKU (16 F-14 M;1–36 year) and 50 healthy volunteers (24 F-26 M; 0–39 year). 17 patients with PKU followed Phe-free AA mixtures without carnitine and the rest had Phe-free protein subtitutes with supplemented carnitine.	MS/MS (Targeted analysis)	Dried blood spot (Fasting)	Not reported	Carnitine Octanoylcarnitine Decanoylcarnitine
		aa analyzer with post-column derivatisation with ninhydrine (Biotronic LC 3000) (Targeted analysis)	Serum (Fasting)	Phenylalanine	Tyrosine Methionine
Xiong et al. (2015)	47 children with PKU and 47 age-matched non-PKU controls (30–60 days). Diet treatment not reported in the study.	GC-MS (Targeted analysis)	Urine (Unspecified)	Phenylpyruvic acid Phenylacetic acid Phenyllactic acid 2-Hydroxyphenylacetic acid Phenylacetylglutamine Phenylalanine Mandelic acid N-Acetylphenylalanine 4-Hydroxyphenylacetic acid 4-Hydroxyphenylpyruvic acid 4-Hydroxyphenyllactic acid	Not reported

^a^Top-25 metabolites reported are shown. aa, amino acids; AA-MF, amino acids medical foods; ED, electrochemical detection; F, female; GC-MS, gas chromatography coupled to mass spectrometry; GMP-MF, glycomacropeptide medical foods; GPI, glycerophosphoinositol; HILIC-MS/MS, hydrophilic interaction liquid chromatography-tandem mass spectrometry; HPA, hyperphenylalaninemia; HPLC, high-performance liquid chromatography; LC-MS/MS, liquid chromatography-tandem mass spectrometry; M, male; NMR, nuclear magnetic resonance; P_3_, percentile 3; P_97_, percentile 97; Phe, phenylalanine; PKU, phenylketonuria; PUFA, polyunsaturated fatty acids; QQQ, triple quadropole; QTOF, quadrupole time-of-flight; RP/UPLC-MS/MS, reverse phase ultra-performance liquid chromatography-tandem mass spectrometry; wk, week; yr, years

#### Studies participants

**Fig. 3:**
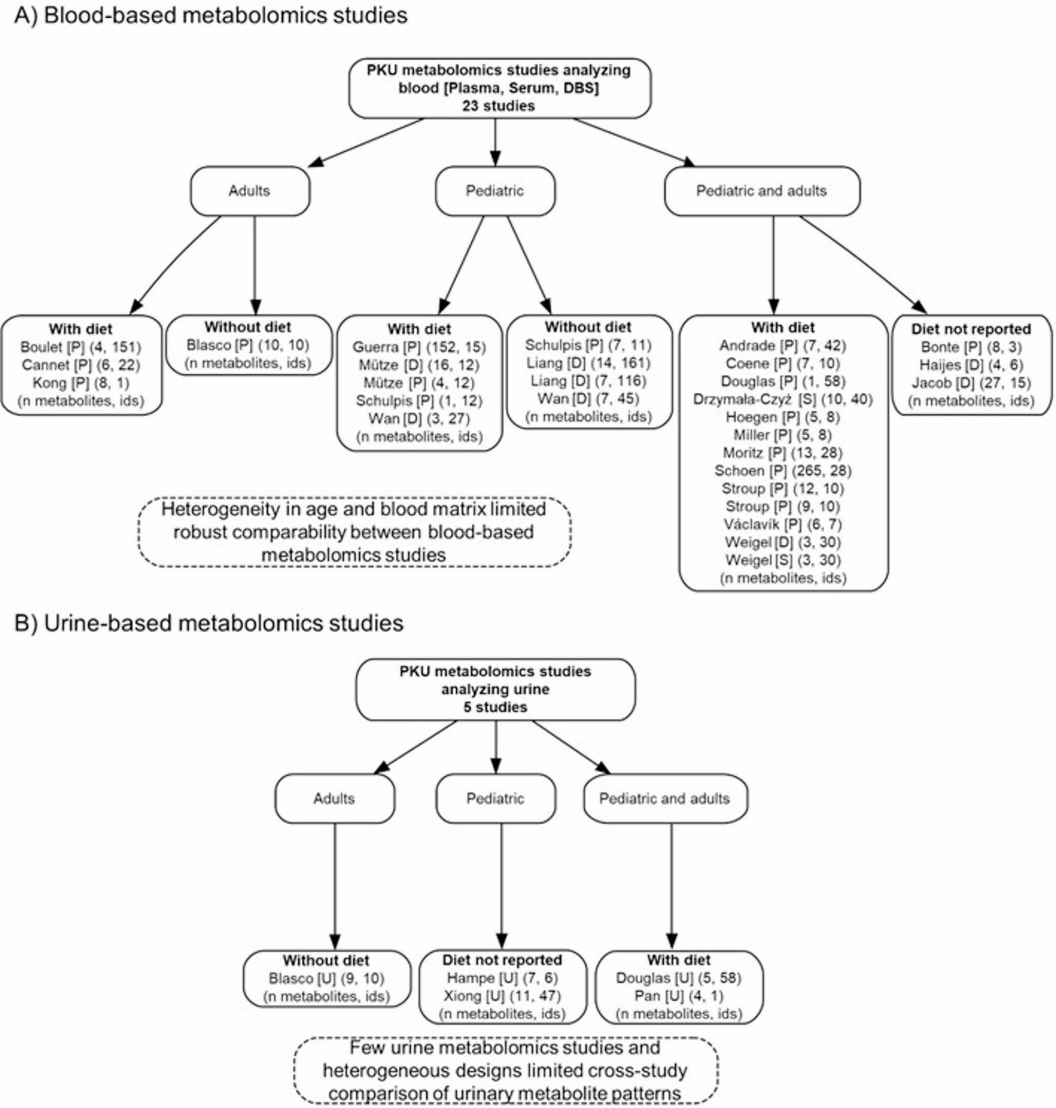
Overview of included PKU metabolomics studies stratified by sample type, age group, and dietary status. D, dried blood spot; ids, number of individuals; P, plasma; S, serum; U, urine

All of the selected studies compared participants with PKU to a healthy control group (Table 1). Figure 3 provides a visual summary of the included studies, illustrating the variability in sample type, age group and dietary treatment across blood- and urine-based metabolomics analyses.

The ages of patients with PKU varied widely, ranging from neonates (Wan et al., 2022) to 57 years (Schoen & Singh, 2022). Seven studies exclusively analyzed pediatric individuals with PKU, four studies focused on PKU adults, and 15 studies. included both groups but did not report results separately by age (Fig. 3). Regarding the sex of the participants, 14 of the 26 studies included both male and female patients without stratifying results by sex (Andrade et al., 2017; Blasco et al., 2017; Boulet et al., 2020; Cannet et al., 2020; Douglas et al., 2013; Drzymała-Czyż et al., 2018; Guerra et al., 2021; Jacob et al., 2018; Liang et al., 2020; Moritz et al., 2023; Mütze et al., 2012; Stroup et al., 2018; Wan et al., 2022; Weigel et al., 2008). Alternatively, other studies focused exclusively on females (Schoen & Singh, 2022) or males (Kong & Hernandez-Ferrer, 2019) while others did not specify the gender of their participants (Bonte et al., 2019; Coene et al., 2018; Haijes et al., 2019; Hampe et al., 2017; Hoegen et al., 2022; Miller et al., 2015; Pan et al., 2007; Schulpis et al., 2002; Václavík et al., 2018; Xiong et al., 2015). The number of patients with PKU by study ranged from one (Kong & Hernandez-Ferrer, 2019; Pan et al., 2007) to 161 (Liang et al., 2020). Differences in dietary treatment were also reported as some participants were not following a dietary treatment at the time of inclusion (without diet), while some studies did not clarify if their individuals with PKU were on dietary treatment (diet unclear) (Fig. 3). Several studies included participants with good dietary adherence (Andrade et al., 2017; Coene et al., 2018; Drzymała-Czyż et al., 2018; Guerra et al., 2021; Hoegen et al., 2022; Kong & Hernandez-Ferrer, 2019; Mütze et al., 2012; Pan et al., 2007; Schulpis et al., 2002; Stroup et al., 2018; Václavík et al., 2018; Wan et al., 2022; Weigel et al., 2008) while other studies had participants with varying adherence to their prescribed dietary treatment (Boulet et al., 2020; Cannet et al., 2020; Douglas et al., 2013; Miller et al., 2015; Moritz et al., 2023; Schoen & Singh, 2022). Additionally, some participants received pharmacotherapies alongside their dietary treatment (Andrade et al., 2017; Schoen & Singh, 2022; Stroup et al., 2018). Further details are provided in Table 1.

#### Biosamples and metabolomic techniques

Almost 90% of the studies analyzed plasma, serum, and DBS samples (Table 1; Fig. 3A) whereas only five studies reported metabolites in urine (Table 1; Fig. 3B). Additional details on sample types and their collection are provided in Table 1.

The studies employed a wide range of metabolomic approaches, leading to considerable methodological variability. Gas chromatography-based approaches were common, including gas chromatography coupled to mass spectrometry (GC-MS) (Blasco et al., 2017; Drzymała-Czyż et al., 2018; Guerra et al., 2021; Hampe et al., 2017; Xiong et al., 2015) and GC with flame ionization (GC-FID) (Mütze et al., 2012). NMR (Blasco et al., 2017; Cannet et al., 2020; Pan et al., 2007) and amino acid analyzers were also utilized (Blasco et al., 2017; Douglas et al., 2013; Mütze et al., 2012; Schulpis et al., 2002; Weigel et al., 2008). Among liquid chromatography-based methods, both hydrophilic interaction liquid chromatography coupled to tandem mass spectrometry approaches (HILIC-MS/MS) (Guerra et al., 2021; Kong & Hernandez-Ferrer, 2019; Schoen & Singh, 2022; Stroup et al., 2018) and reverse-phase liquid chromatography with mass spectrometry (RP-LC-MS) were frequently employed (Andrade et al., 2017; Bonte et al., 2019; Boulet et al., 2020; Coene et al., 2018; Hoegen et al., 2022; Jacob et al., 2018; Kong & Hernandez-Ferrer, 2019; Miller et al., 2015; Moritz et al., 2023; Schoen & Singh, 2022; Stroup et al., 2018; Václavík et al., 2018). Additional techniques included flow injection analysis (Mütze et al., 2012), direct infusion MS (Haijes et al., 2019), direct injection MS (Liang et al., 2020), electrochemical detection (Douglas et al., 2013; Schulpis et al., 2002), desorption electrospray ionization (Pan et al., 2007), as well as unspecified LC-MS/MS or MS/MS approaches (Wan et al., 2022; Weigel et al., 2008). Further methodological details are provided in Table 1.

Given the methodological variability across studies, including differences in sample handling, metabolomic platforms and quantification strategies, we considered plasma, serum and DBS collectively as blood-derived matrices, allowing for a more consistent and comparable interpretation of result.

### Quality assessment of the included studies using QUADOMICS

The 70% cut-off score applied in this systematic review was met by all the included studies. From the eight items of the adapted QUADOMICS tool (Carrard et al., 2022; Lumbreras et al., 2008), all studies fulfilled the following item: reporting intermediate or uninterpretable results (Table 1; Supplementary Table 3). Several studies did not report either the age, the gender or the presence of dietary treatment, therefore they did not get 1 point in item 1 (Bonte et al., 2019; Coene et al., 2018; Haijes et al., 2019; Hampe et al., 2017; Hoegen et al., 2022; Jacob et al., 2018; Kong & Hernandez-Ferrer, 2019; Miller et al., 2015; Pan et al., 2007; Schulpis et al., 2002; Václavík et al., 2018; Xiong et al., 2015). Most studies (Andrade et al., 2017; Blasco et al., 2017; Boulet et al., 2020; Cannet et al., 2020; Douglas et al., 2013; Drzymała-Czyż et al., 2018; Guerra et al., 2021; Liang et al., 2020; Mütze et al., 2012; Schoen & Singh, 2022; Schulpis et al., 2002; Stroup et al., 2018; Wan et al., 2022; Weigel et al., 2008) analyzed fasting samples. Moreover, nine studies did not mention the fasting status of their samples (Haijes et al., 2017; Hampe et al., 2017; Jacob et al., 2018; Kong & Hernandez-Ferrer, 2019; Miller et al., 2015; Moritz et al., 2023; Pan et al., 2007; Václavík et al., 2018; Xiong et al., 2015) and three studies did not follow a protocol regarding time of specimen collection (Bonte et al., 2019; Coene et al., 2018; Hoegen et al., 2022). Three studies did not provide sufficient detail or cite a clear reference regarding the metabolomic technique (Schulpis et al., 2002; Wan et al., 2022; Weigel et al., 2008). Following the recommendations by Parker et al. (2010), the 11th item was scored with 1 if the diagnostic process was described in sufficient detail (Parker et al., 2010), and 14 studies (Andrade et al., 2017; Bonte et al., 2019; Boulet et al., 2020; Cannet et al., 2020; Coene et al., 2018; Drzymała-Czyż et al., 2018; Guerra et al., 2021; Hoegen et al., 2022; Jacob et al., 2018; Liang et al., 2020; Miller et al., 2015; Moritz et al., 2023; Wan et al., 2022; Xiong et al., 2015) completely accomplish this criterion. Item 16 (Supplementary Table 2) was only applicable to Blasco et al. (2017), as they created a multivariate model for their study (Blasco et al., 2017). However, they used an internal validation through cross-validation rather than using an independent set of patient samples (Parker et al., 2010). Therefore, we did not consider item 16 in Supplementary Table 3, as the other studies did not address overfitting in their analyses. Further information is available in Supplementary Table 3.

### Metabolomic profiling in phenylketonuria

A total of 544 metabolites that differed between patients with PKU and HC were identified in the 26 selected studies. Of these, 517 metabolites were described in plasma, serum or DBS, while only 27 metabolites were reported in urine samples. The complete list of metabolites reported in urine and blood—together with their identifiers from the Human Metabolome Database (HMDB) (Wishart et al., 2022), PubChem, KEGG and SMILES annotation; the reference author; the frequency of reporting across studies; the type of blood matrix analyzed (plasma, serum, or DBS); and the metabolic pathways assigned by HMDB and by MetaboAnalyst (via the KEGG database)—is provided in Supplementary Table 4. Additionally, for urinary metabolites, we indicate whether their changes were consistent with the corresponding findings in blood. Among the 27 urinary metabolites identified, 14 were also reported in blood and 10 of these exhibited changes in the same direction.

#### Metabolomic fingerprinting in blood samples

**Fig. 4:**
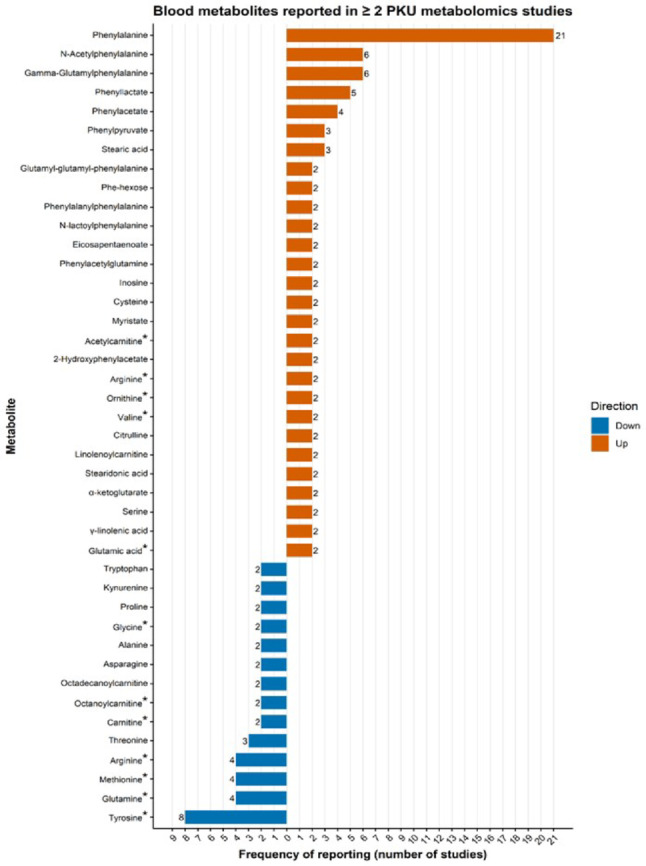
Blood metabolites consistently reported in two or more PKU metabolomics studies in the same direction. *for metabolites reported in both directions across studies. Arginine was reported 2 ≥ times in both directions (see Table 2)

Most included studies analyzed blood-derived matrices, including plasma, serum and DBS (Fig. 3). Across these studies, a total of 517 metabolites were reported as significantly altered in individuals with PKU compared with healthy controls, of which 312 were upregulated and 205 were downregulated (Supplementary Table 4). Due to the heterogeneity in analytical platforms and metabolomics approaches, the majority of metabolites were only identified in a single study. Only 41 metabolites were consistently reported two or more times in the same direction (i.e., up- or downregulated; Fig. 4).

**Fig. 5:**
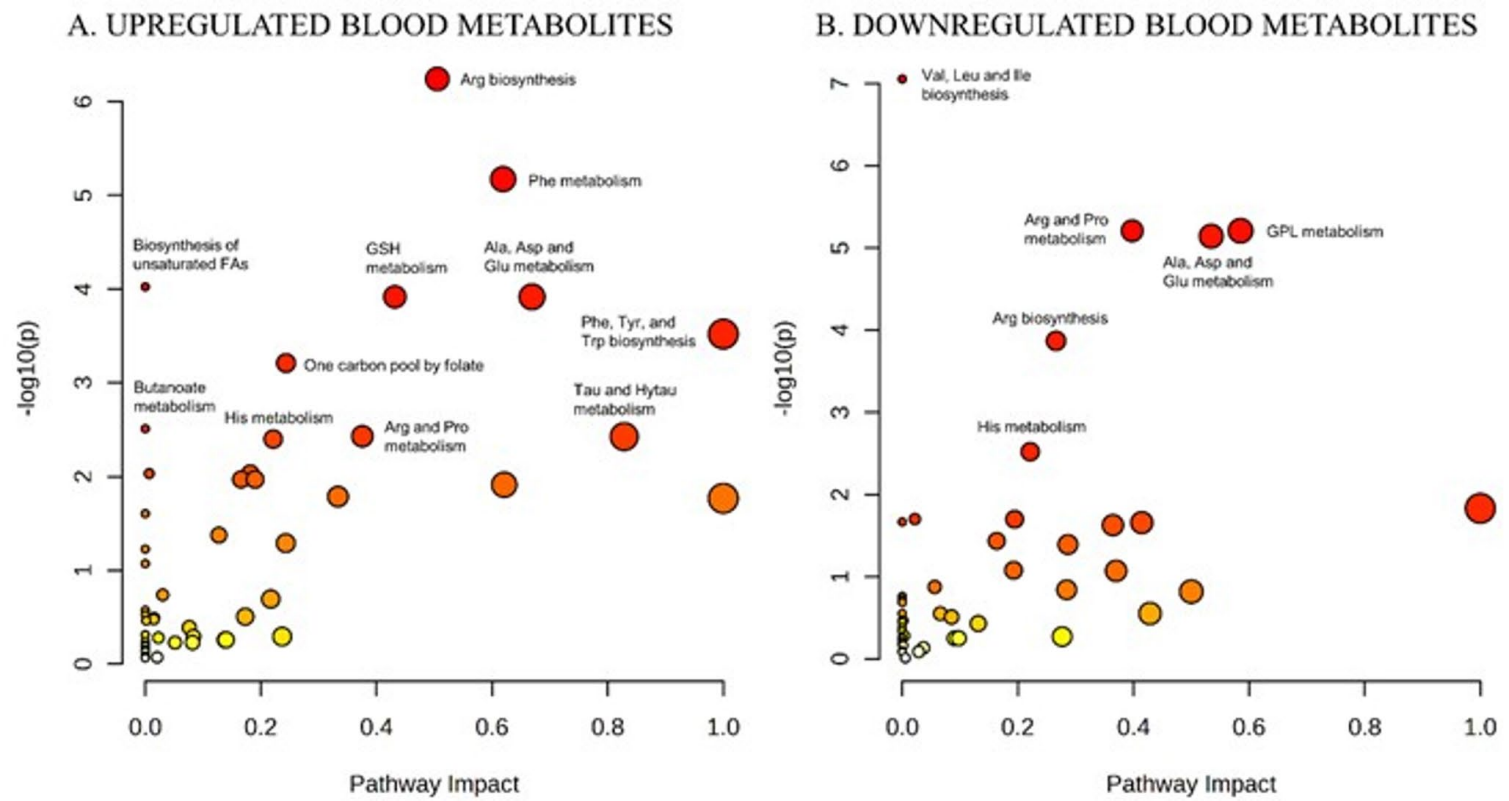
Pathway impact of up- **A** and downregulated **B** blood metabolites. The color of the circle represents the significance level in the enrichment analysis, with red indicating higher significance and yellow indicating lower significance. The size of the circle corresponds to the pathway impact value in the topology analysis, with larger circles indicating greater impact. The x-axis displays the pathway impact value calculated from the topology analysis. Ala, alanine; Arg, arginine; Asp, aspartate; FA, fatty acid; Glu, glutamate; GPL, glycerophospholipid; GSH, glutathione; His, histidine; Hytau, hypotaurine; Ile, isoleucine; Leu, leucine; Phe, phenylalanine; Pro, proline; Tau, taurine; Trp, tryptophan; Tyr, tyrosine; Val, valine

As expected, Phe was the most frequently reported upregulated metabolite, appearing in 21 studies, and several Phe-derived metabolites (e.g., N-acetylphenylalanine, γ-glutamylphenylalanine, phenyllactate) were also consistently elevated across studies. The upregulated levels of Phe, phenylpyruvate, and phenylacetate, among others, contributed to the significant alteration of the Phe metabolism pathway (FDR 2.68 × 10⁻⁴; impact = 0.62) (Fig. 5A; Supplementary Table 5).

Beyond Phe, the upregulation of several amino acids led to the enrichment of additional pathways, including alanine (Ala), aspartate (Asp) and glutamate (Glu) metabolism, taurine and hypotaurine metabolism, histidine (His) metabolism, and arginine (Arg) and proline (Pro) metabolism. Notably, Arg biosynthesis emerged as the most statistically significant pathway (4.57 × 10⁻⁵), driven by increased levels of Arg, as well as other related amino acids such as glutamine (Gln), ornithine and citrulline. Furthermore, elevated levels of other compounds, including glutathione (GSH) and several fatty acids (FA), contributed to the enrichment of GSH metabolism and the biosynthesis of unsaturated fatty acids, respectively. Conversely, six pathways were significantly downregulated (FDR < 0.05; Fig. 5B; Supplementary Table 6). Among these, glycerophospholipid (GPL) metabolism showed the highest pathway impact (0.59) due to the downregulation of key phosphatidylethanolamine and phosphocholine metabolites. Valine, leucine and isoleucine biosynthesis was the most statistically significant downregulated pathway (7.04 × 10⁻^6^). Interestingly, the four remaining downregulated pathways (Arg and Pro metabolism; Ala, Asp and Glu metabolism; Arg biosynthesis and His metabolism) displayed both up- and downregulated metabolites across studies (Fig. 5; Supplementary Tables 5–6). In these sense, a total of 35 metabolites were reported to be both up- and downregulated across 14 studies (Andrade et al., 2017; Blasco et al., 2017; Cannet et al., 2020; Drzymała-Czyż et al., 2018; Guerra et al., 2021; Haijes et al., 2019; Jacob et al., 2018; Liang et al., 2020; Moritz et al., 2023; Mütze et al., 2012; Schoen & Singh, 2022; Schulpis et al., 2002; Wan et al., 2022; Weigel et al., 2008), 24 of which were reported only twice but in opposite directions (Table 2).

**Table 2:** Blood metabolites concurrently upregulated and downregulated in PKU vs. CON

Metabolite	[↑] PKU vs. CON	[↓] PKU vs. CON	Interpretation of findings
Tyrosine	Moritz et al. (2023)	Cannet et al. (2020) Weigel et al. (2008) Liang et al. (2020) Blasco et al. (2017) Schoen and Singh (2022) Wan et al. (2022) Schulpis et al. (2002) Haijes et al. (2019)	Differences could be attributed to the supplementation with medical food and plasma levels varying with respect to the dose and frequency of Tyr supplementation (Moritz et al., 2023). Normally, Tyr should be down or in normal ranges when strictly adhered to dietary treatment or in lower ranges when under poor metabolic control.
Citric acid	Cannet et al. (2020)	Moritz et al. (2023)	Differences could be attributed to the adherence to the PKU dietary treatment and metabolic control (Cannet et al., 2020; Moritz et al., 2023).
Glutamic acid	Cannet et al. (2020) Moritz et al. (2023)	Liang et al. (2020)	Differences could be attributed to the adherence to the PKU dietary treatment (Cannet et al., 2020; Moritz et al., 2023) versus pre-therapeutic subjects (Liang et al., 2020). The use of different blood-matrices (DBS or plasma) might also explain these differences.
Aspartate	Moritz et al. (2023)	Liang et al. (2020)	
Methionine sulfoxide	Moritz et al. (2023)	Schoen and Singh (2022)	Differences could be attributed to the PKU dietary treatment (Moritz et al., 2023) or the gender of the participants (Schoen & Singh, 2022).
Ornithine	Liang et al. (2020) Andrade et al. (2017)	Liang et al. (2020)	Differences could be attributed to the age, mean of 5 days versus 3 months of life (Liang et al., 2020) versus children and adults (Andrade et al., 2017), and the impact of not following any treatment during a prolonged period (Liang et al., 2020). The use of different blood-matrices (DBS or plasma) might also explain these differences.
Histidine	Liang et al. (2020)	Schoen and Singh (2022)	Differences could be attributed to the adherence to the PKU dietary treatment (Schoen & Singh, 2022) versus pre-therapeutic subjects (Liang et al., 2020). The use of different blood-matrices (DBS or plasma) might also explain these differences.
Methionine	Wan et al. (2022)	Weigel et al. (2008) Liang et al. (2020) Blasco et al. (2017) Schoen and Singh (2022)	Differences could be attributed to the age, mean of 2 weeks (Wan et al., 2022) versus 3 months of life (Liang et al., 2020) versus pediatric and adults (Andrade et al., 2017; Jacob et al., 2018; Schoen & Singh, 2022; Weigel et al., 2008), to the no Phe-restricted diet patients (Blasco et al., 2017), not reported dietary treatment (Jacob et al., 2018), or to the adherence to the PKU dietary treatment (Andrade et al., 2017; Schoen & Singh, 2022; Weigel et al., 2008). The use of different blood-matrices (DBS or plasma) might also explain these differences.
Arginine	Wan et al. (2022) Jacob et al. (2018)	Liang et al. (2020) Blasco et al. (2017) Schoen and Singh (2022) Andrade et al. (2017)	
Hydroxybutyrylcarnitine	Schoen and Singh (2022)	Mütze et al. (2012)	Differences in carnitine’s levels could be attributed to the adherence to the PKU dietary treatment (Schoen & Singh, 2022), not reported dietary treatment (Jacob et al., 2018), or metabolic disturbances caused by PKU (Mütze et al., 2012; Weigel et al., 2008). The use of different blood-matrices (DBS or plasma) might also explain these differences.
Acetylcarnitine	Schoen and Singh (2022) Jacob et al. (2018)	Mütze et al. (2012)	
Hexanoylcarnitine	Schoen and Singh (2022)	Mütze et al. (2012)	
Octanoylcarnitine	Schoen and Singh (2022)	Mütze et al. (2012) Weigel et al. (2008)	
Decanoylcarnitine	Schoen and Singh (2022)	Weigel et al. (2008)	
Linoleate	Schoen and Singh (2022)	Drzymała-Czyż et al. (2018)	Differences in the lipid profile could be attributed to the adherence to the PKU dietary treatment (Drzymała-Czyż et al., 2018; Schoen & Singh, 2022) or metabolic disturbances caused by PKU (Drzymała-Czyz et al., 2018). The use of different blood-matrices (plasma or serum) might also explain these differences.
Adrenate	Schoen and Singh (2022)	Drzymała-Czyż et al. (2018)	
Docosapentaenoic acid	Schoen and Singh (2022)	Drzymała-Czyż et al. (2018)	
Mead Acid	Schoen and Singh (2022)	Drzymała-Czyż et al. (2018)	
Docosahexaenoic acid	Guerra et al. (2021)	Drzymała-Czyż et al. (2018)	Differences in the lipid profile could be attributed to the adherence to the PKU dietary treatment (Drzymała-Czyż et al., 2018; Schoen & Singh, 2022) and the use of PUFA supplements (Guerra et al., 2021). The use of different blood-matrices (plasma or serum) might explain the differences of docosahexaenoic acid levels.
SM(d18:1/16:0)	Guerra et al. (2021)	Schoen and Singh (2022)	
LPC(18:0)	Guerra et al. (2021)	Schoen and Singh (2022)	
LPC(16:1)	Guerra et al. (2021)	Schoen and Singh (2022)	
LPC(16:0)	Guerra et al. (2021)	Schoen and Singh (2022)	
SM(d18:1/20:0)	Guerra et al. (2021)	Schoen and Singh (2022)	
LPC(20:4)	Guerra et al. (2021)	Schoen and Singh (2022)	
LPC(18:2)	Guerra et al. (2021)	Schoen and Singh (2022)	
LPC(18:1)	Guerra et al. (2021)	Schoen and Singh (2022)	
Glutathione	Jacob et al. (2018)	Moritz et al. (2023)	Differences levels could be attributed to the adherence to the PKU dietary treatment (Moritz et al., 2023; Cannet et al., 2020) or not reported dietary treatment (Jacob et al., 2018). The use of different blood-matrices (DBS or plasma) might also explain these differences.
Taurine	Moritz et al. (2023)	Jacob et al. (2018)	
Hydroxyproline	Jacob et al. (2018)	Mütze et al. (2012)	
Creatinine	Jacob et al. (2018)	Cannet et al. (2020)	
Carnitine	Jacob et al. (2018)	Mütze et al. (2012) Weigel et al. (2008)	Differences in carnitine levels could be attributed to the adherence to the PKU dietary treatment, or not reported dietary treatment (Jacob et al., 2018) or metabolic disturbances caused by PKU (Mütze et al., 2012; Weigel et al., 2008).
Valine	Liang et al. (2020) Wan et al. (2022)	Jacob et al. (2018)	Differences in valine levels may be attributed to the age, newborns and young children (Liang et al., 2020; Wan et al., 2022), versus children and adults (Jacob et al., 2018).
Glycine	Andrade et al. (2017)	Liang et al. (2020) Jacob et al. (2018)	Differences in glycine levels may be attributed to the age, 3 months of life (Liang et al., 2020) versus pediatric and adults (Andrade et al., 2017; Jacob et al., 2018), PKU dietary adherence (Andrade et al., 2017) or not reported dietary treatment (Jacob et al., 2018). The use of different blood-matrices (DBS or plasma) might also explain these differences.
Glutamine	Jacob et al. (2018)	Cannet et al. (2020) Liang et al. (2020) Schoen and Singh (2022) Blasco et al. (2017)	Differences in glutamine levels might be attributed to the different cohort characteristics and dietary adherence (Cannet et al., 2020; Schoen & Singh, 2022), to the no Phe-restricted diet patients (Blasco et al., 2017) and not reported dietary treatment (Jacob et al., 2018). The use of different blood-matrices (DBS or plasma) might also explain these differences.

CON, control population, LPC, lysophosphatidylcholine, Phe, phenylalanine, PKU, phenylketonuria, PUFA, polyunsaturated fatty acid, SM, sphingomyelin, Tyr, tyrosine

Overall, the heterogeneous pattern observed across metabolites that were reported as both up- and downregulated reflects substantial differences among the included studies (Fig. 3). Variability observed in cohort characteristics such as age (ranging from newborns to adults) or gender, differences in dietary adherence with the presence or absence of a Phe-restricted diet, as well as the use of specific supplements (e.g., Tyr or polyunsaturated fatty acids (PUFA)), all contributing to these observed differences. In addition, differences in analytical platforms, metabolomics approaches, and the use of distinct blood matrices (plasma, serum, DBS) may explain the lack of consistency across studies in the regulation of several metabolites, including amino acids, carnitine compounds and lipid-related compounds.

#### Metabolomic fingerprinting in blood of diet-treated individuals with phenylketonuria

To specifically assess the metabolic fingerprint in diet-treated individuals with PKU, we focused on studies that included participants with dietary treatment. However, dietary reporting varied considerably across studies, with some providing limited or unspecific descriptions of dietary patterns, protein substitutes, and additional supplementation.

Amino acids compounds were the most frequently reported alterations across studies compared with healthy controls (Tables 1 and 2). Among studies including participants on a Phe-restricted diet, changes were reported for Glu, Tyr, Asp, Arg, among other amino acids. Notably, Arg was reported as downregulated both in cohorts with good adherence (Andrade et al., 2017) and in cohorts with poorer adherence (Schoen & Singh, 2022), suggesting that its direction of change may not be explained by diet alone. Another metabolite likely influenced by dietary factors is tyrosine, which was unexpectedly reported as upregulated in Moritz et al. (2023) and could reflect additional Tyr supplementation in some participants. However, time of sample collection was not clearly reported in this study (Moritz et al., 2023). Overall, these findings suggest that diet composition, supplementation, and adherence can influence amino acid-related metabolites in PKU, highlighting the need for future studies with better dietary characterization and stratified analyses to clarify these effects and their clinical implications.

Carnitine-related metabolites were also altered, with lower levels of several acylcarnitines reported in cohorts adhering to a Phe-restricted diet (Mütze et al., 2012; Weigel et al., 2008), whereas elevated levels were observed in cohorts with poor dietary adherence (Schoen & Singh, 2022) (Table 2). In addition, free carnitine was reduced in the diet-treated cohorts, but was reported as upregulated in the cohort described by Jacob et al. (2018), although dietary treatment was not specified in that study. These findings may reflect an increased requirement for carnitine supplementation, particularly in individuals with PKU who have higher metabolic demands (Weigel et al., 2008).

Differences in lipid-related metabolites were consistently observed across studies (Table 2). For example, docosahexaenoic acid (DHA) was reported as upregulated in children with PKU in the study by Guerra et al. (2021), whereas it was downregulated in another cohort including both children and adults under good metabolic control (Drzymała-Czyż et al., 2018). These contrasting findings may reflect differences in PUFA supplementation, which was implemented in some individuals of the cohort studied by Guerra et al. (2021), as well as broader metabolic alterations associated with PKU (Drzymała-Czyż et al., 2018). Similarly, discrepancies in sphingomyelin and lysophosphatidylcholine species were observed between the cohorts reported by Schoen and Singh (2022) and Guerra et al. (2021). These differences may be partially explained by variations in treatment strategies, as some participants in the study by Schoen and Singh consumed glycomacropeptide medical foods, and others were treated with pegvaliase or sapropterin dihydrochloride, whereas Guerra et al. (2021) studied children on a life-long Phe-restricted diet with essential amino acid formulas and PUFA supplementation. Notably, more than half of the participants in the cohort described by Schoen and Singh did not fully adhere to their prescribed dietary treatment, which may have further contributed to the observed variability in lipid profiles. In addition, other FAs were also altered (Table 2), showing opposite directions between cohorts with varying dietary adherence (Schoen & Singh, 2022) and cohorts with good metabolic control (Drzymała-Czyż et al., 2018). These differences may reflect the impact of dietary treatment, although they could also be influenced by using different metabolomic techniques and blood matrices across studies.

#### Metabolomic fingerprint in urine samples

**Fig. 6:**
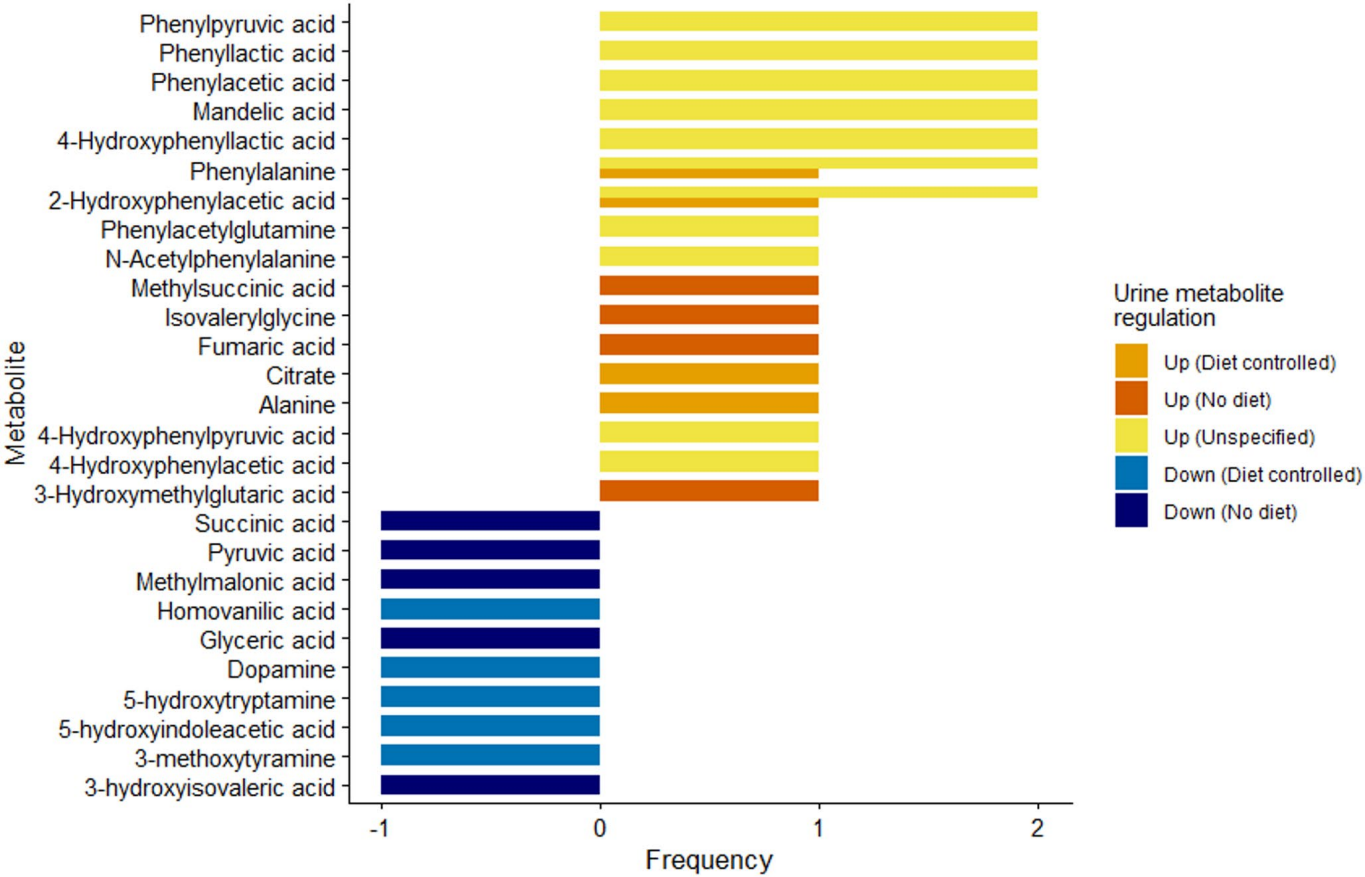
Up- and downregulated urinary metabolites across PKU studies

A total of 27 urinary metabolites were reported as altered in patients with PKU compared with healthy controls across five studies (Blasco et al., 2017; Douglas et al., 2013; Hampe et al., 2017; Pan et al., 2007; Xiong et al., 2015), of which 17 were upregulated (Fig. 6). Most of these metabolites correspond to Phe and Phe-derived compounds, reflecting the metabolic overflow caused by Phe accumulation in PKU similar than in blood (Rausell et al., 2019; Xiong et al., 2015). This includes phenylpyruvic acid, phenylacetic acid, and 2-hydroxyphenylacetic acid, among others, which were consistently reported in at least two GC/MS studies (Hampe et al., 2017; Xiong et al., 2015). Pan et al. (2007) additionally proposed Ala and citrate as potential urinary biomarkers; however, their study included only a single patient with PKU, and the inconsistent results observed in blood metabolomics studies (Cannet et al., 2020; Moritz et al., 2023; Liang et al., 2020; Blasco et al., 2017) suggest that these findings should be interpreted with caution and require validation in larger cohorts.

Regarding the 10 downregulated urinary metabolites (Fig. 6), Douglas et al. (2013) reported lower baseline concentrations of several urinary monoamines (Table 1), which play key roles in neurotransmitter metabolism. In their study, plasma Phe levels showed an inverse association with these markers, underscoring the importance of maintaining adequate dietary control (Douglas et al., 2013). Lastly, Blasco et al. (2017) observed reduced levels of several organic acids (Table 1), but their cohort did not follow a Phe-restricted diet, which complicates comparison with diet-controlled studies (Blasco et al., 2017).

## Discussion

To our knowledge, this is the first systematic review to comprehensively evaluate metabolomic alterations reported in 26 studies, encompassing a total of 544 metabolites identified in blood and urine samples (517 blood-derived and 27 urine-derived), in comparison with control groups.

Phe was consistently found to be highly elevated in patients with PKU across 21 blood-based studies (Fig. 4; Andrade et al., 2017; Blasco et al., 2017; Bonte et al., 2019; Boulet et al., 2020; Cannet et al., 2020; Coene et al., 2018; Douglas et al., 2013; Haijes et al., 2019; Hoegen et al., 2022; Jacob et al., 2018; Kong & Hernandez-Ferrer, 2019; Liang et al., 2020; Miller et al., 2015; Moritz et al., 2023; Mütze et al., 2012; Schoen & Singh, 2022; Schulpis et al., 2002; Stroup et al., 2018; Václavík et al., 2018; Wan et al., 2022; Weigel et al., 2008), together with several Phe-derivates (e.g., phenyllactate, phenylacetate, N-acetylphenylalanine and γ-glutamylphenylalanine). Elevated Phe levels were also reported in three urine-based studies (Hampe et al., 2017; Pan et al., 2007; Xiong et al., 2015).

In contrast, Tyr levels were generally downregulated in most studies (Blasco et al., 2017; Cannet et al., 2020; Haijes et al., 2019; Liang et al., 2020; Schoen & Singh, 2022; Schulpis et al., 2002; Wan et al., 2022; Weigel et al., 2008) or remained within the normal range (Mütze et al., 2012; Wan et al., 2022) consistent with PAH deficiency. However, in treated patients, Moritz et al. (2023) reported higher Tyr levels compared with healthy individuals (Moritz et al., 2023). This increase could be attributable to the composition of protein substitutes and Tyr supplementation, with additional variability potentially arising from differences in supplementation regimens (dose and frequency) and sampling time (Moritz et al., 2023). Elevated Phe levels have been related to neurophysiological and neuropsychological dysfunction (Dobrowolski et al., 2022) likely through multiple mechanisms, including impaired protein synthesis, myelin damage, and reduced neurotransmitter synthesis, among others (Blau et al., 2010; de Groot et al., 2015; van Wegberg et al., 2017). Phe entry into the brain is mediated by the large neutral amino acid (LNAA) type-1 transporter (LAT1), where it competes with other LNAAs for transport. Phe appears to have a higher affinity for LAT1 compared to other LNAAs, resulting in elevated brain Phe concentrations and reduced concentrations of other LNAAs (de Groot et al., 2015; van Wegberg et al., 2017). Consequently, levels of Tyr and Trp, two key LNAAs in the brain, may be decreased (Rausell et al., 2019; van Spronsen et al., 2021), hypothetically leading to deficiencies in dopamine and serotonin (Fig. 1), respectively. Notably, both dopamine and serotonin were reported to be downregulated in both blood (Schulpis et al., 2002) and urine samples (Douglas et al., 2013). This deficiency is probably linked to mood disorders and the high prevalence of anxiety observed in PKU patients (van Spronsen et al., 2021). In this context, Boulet et al. (2020) reported alterations in tryptophan (Trp) metabolism in treated patients with PKU, noting that kynurenine (Kyn), a metabolite whose pathway accounts for approximately 95% of Trp catabolism, was downregulated despite normal Trp levels compared to healthy controls. However, when participants with PKU were stratified by dietary treatment (with or without a controlled low-Phe diet) and by supplementation with amino acid formulas, no differences in Trp and Kyn levels were observed. In addition, lower levels of 3-hydroxykynurenic acid (3HK), a precursor of the neurotoxic quinolinic acid, were found in patients with PKU compared to healthy individuals (Boulet et al., 2020). The study by Schoen and Singh (2022), observed lower levels of both plasma Trp and Kyn, but no differences on 3HK (Schoen & Singh, 2022). Moreover, Gassió et al. (2019) reported reduced platelet serotonin concentrations in some patients with PKU (Gassió et al., 2019). These findings suggest the need for further investigation, as Trp metabolism is clearly affected in PKU.

Another potential alteration in the brain is the reduction in Glu synaptic transmission (Dobrowolski et al., 2022; Rausell et al., 2019; van Wegberg et al., 2017), which may be influenced by disturbances in Glu and Gln metabolism. Glu plays a critical role as both a neurotransmitter (Hertz, 2013) and a regulator of ammonia homeostasis within the central nervous system. Additionally, Glu is essential for energy metabolism through the TCA cycle (Schousboe et al., 2014). Across studies, Glu levels were found to be upregulated in adult patients with a Phe-restricted diet (Cannet et al., 2020; Moritz et al., 2023) but downregulated in pre-therapeutic children (Liang et al., 2020). In contrast, Gln was consistently downregulated (Blasco et al., 2017; Cannet et al., 2020; Liang et al., 2020; Schoen & Singh, 2022) except for the study of Jacob et al. (2018), which did not mention the dietary treatment of their cohort. Differences in sample type, analytical methodology, and cohort characteristics may account for this between-study variability. Gln depletion in PKU has been a topic of discussion for decades, as it may impair brain development (Perry et al., 1970). Recent findings, such as elevated urinary phenylacetylglutamine, may provide new insights into Gln metabolism in PKU pathophysiology (Andrade et al., 2021; Xiong et al., 2015). However, these findings highlight the need for further research, as Glu and Gln metabolism in the brain is exceedingly complex.

Another key aspect frequently reported in the literature is increased oxidative stress, which may play a role in the pathophysiology of PKU (Dobrowolski et al., 2022; Ribas et al., 2011; Sanayama et al., 2011; Sirtori et al., 2005). Arg, a precursor of nitric oxide (Fig. 1) that is involved in oxidative stress pathways, has been reported to be predominantly downregulated (Table 2) (Andrade et al., 2017; Blasco et al., 2017; Liang et al., 2020; Schoen & Singh, 2022), although some studies have observed upregulated levels (Jacob et al., 2018; Wan et al., 2022). Furthermore, Moritz et al. (2023) observed decreased levels of GSH metabolites, suggesting that oxidative stress in patients with PKU may be altered (Moritz et al., 2023), although Jacob et al. (2018) found it to be upregulated. Additional metabolomics research is needed to clarify these results. Sanayama et al. (2011) reported significantly lower plasma total antioxidant reactivity levels in patients with PKU, with oxidative stress closely linked to serum Phe levels (Sanayama et al., 2011). In contrast, Artuch et al. (2004) did not find a significant increase in oxidative stress. However, they observed a decrease in catalase activity and a tendency toward lower plasma coenzyme Q10 levels with advancing patient age (Artuch et al., 2004). Although micronutrient deficiencies (e.g. selenium, zinc, coenzyme Q10, and L-carnitine) have been linked to oxidative stress, the current European guidelines for PKU do not recommend routine biochemical monitoring of oxidative stress (van Wegberg et al., 2025).

Carnitine, a metabolite with antioxidant properties, can be obtained from both dietary sources and endogenous synthesis in humans. In omnivorous subjects, approximately 75% of carnitine is sourced from dietary intake, primarily from meat, fish, and dairy products, while about 25% is produced endogenously (Li & Zhao, 2021). Free carnitine was observed to be downregulated (Mütze et al., 2012; Weigel et al., 2008) from DBS samples in patients with PKU on a low-Phe diet. Interestingly, Jacob et al. (2018) found higher levels of free carnitine in their cohort but dietary treatment was not reported (Table 2). Free carnitine is commonly found in lower levels in patients with PKU (Sitta et al., 2009; Vilaseca et al., 1993, 2010) and may be a consequence of the strict restriction of foods high in natural protein in this population. Reduced carnitine levels in patients with PKU likely affect its role in transporting long-chain FAs across the inner mitochondrial membrane, which is essential for beta-oxidation and ATP production (Sitta et al., 2009). Therefore, patients with PKU appear to exhibit an altered FA profile. Mütze et al. (2012), reported elevated γ-linolenic acid and normal eicosapentaenoic acid (EPA) and DHA levels (Mütze et al., 2012), while Drzymała-Czyż et al. (2018) found increased levels of both γ-linolenic- and α-linolenic acids, with normal EPA concentrations but diminished DHA levels (Drzymała-Czyż et al., 2018). Both PKU populations were adherent to their low-Phe diet, but no additional PUFA supplementation was reported (Drzymała-Czyż et al., 2018; Mütze et al., 2012). In line with this, Guerra et al. (2021) reported differences in the lipidome of children with PKU, observing increased levels of DHA and EPA as a result of PUFA supplementation (Guerra et al., 2021). They also conducted a review (Guerra et al., 2020) in which they noted that most of studies reported a decrease in DHA, arachidonic acid and EPA. These FAs play critical roles in the central nervous system (Drzymała-Czyż et al., 2018) and are essential components of cell membranes, contributing both structural and functional capabilities. In this context, it is possible that endogenous PUFA synthesis may be impaired, and thus, supplementation has been considered (Drzymała-Czyż et al., 2018; Sanjurjo et al., 1994). Consequently, PUFAs are added to many amino acid formulas and a higher intake of DHA is recommended to women considering and during pregnancy to reduce the risk of preterm birth (van Wegberg et al., 2025).

Mütze et al. (2012) also found decreased hydroxyproline levels in patients with PKU (Mütze et al., 2012), which differ from findings in other studies (Jacob et al., 2018) that reported higher amounts of hydroxyproline. This alteration has been suggested to affect bone metabolism and may partially explain the low bone mineral density reported in this population (Jacob et al., 2018). In addition, Dybal et al. (2025) recently found that the nature of protein intake in dietary treatment may influence bone density in some patients with PKU (Dybal et al., 2025), although further research is needed.

Choline compounds may play a role in the structural integrity and functionality of the brain (Schoen et al., 2022; Schoen & Singh, 2022), and possess potential antioxidant properties (Schoen & Singh, 2022). Schoen and Singh (2022) reported shifts in choline-containing phospholipids in participants with PKU after a 5-day intervention consisting of both nutritional education and diet provision, after which their levels increased from baseline (Schoen & Singh, 2022). Moritz et al. (2023) initially noted that choline levels were at the lower end of the reference range. However, they ultimately discarded it as a strong candidate biomarker due to conflicting results when comparing choline levels with those of healthy individuals (Moritz et al., 2023). Further validation of choline compounds in PKU is needed, and metabolomic studies are essential to clarify their potential as biomarkers.

Finally, dietary adherence is essential for the management of PKU, as it constitutes the primary treatment. It significantly impacts Phe levels and ensures that patients receive adequate nutrition, leading to improved health outcomes (Blau et al., 2010). In this context, Wan et al. (2022) observed alterations in some amino acids (e.g. Arg, methionine, Tyr and Pro) in neonates before treatment (Wan et al., 2022). However, in the 1- to 4-year-old children with PKU group following a Phe-restricted diet upon diagnosis, a complete or partial restoration of these amino acid alterations was observed, underscoring the importance of dietary adherence. Additionally, during the 5-day intervention mentioned before (Schoen & Singh, 2022), a decline in phenylketones was detected, along with a closer alignment with healthy individuals in the abundance of FAs, ketone metabolites, and choline compounds, among others. Wild et al. (2019) compared plasma and urine metabolites between subgroups of patients with PKU based on their dietary control and observed differences in several metabolites. From plasma samples, patients with poor dietary control exhibited higher quantities of Phe and lower levels of Tyr. Increased concentrations of Phe and Phe-derived metabolites from urine samples and decreased carnitine levels were also reported (Wild et al., 2019). Further metabolomic studies evaluating the impact of diet on the metabolome could enhance dietary therapy through a more individualized approach tailored to each patient’s phenotype (Ulaszewska et al., 2019). Given the challenges in achieving complete dietary adherence, especially in adolescence and adulthood, such studies may offer valuable insights for optimizing different treatment strategies to ensure adequate levels of important compounds (Firman et al., 2022; MacDonald et al., 2010).

### Strengths and limitations

To the best of our knowledge, this work represents the first systematic review to comprehensively describe a large number of metabolites exhibiting up- and downregulation of metabolites in PKU, as identified in blood and urine samples analyzed through metabolomic approaches. Consequently, this systematic review provides a comprehensive overview of the current evidence and may guide future studies aiming to characterize the metabolomic fingerprint of PKU.

However, several limitations must be acknowledged. There was considerable heterogeneity in cohorts, blood-derived matrices (plasma, serum, DBS), analytical platforms, sample collection time, and reporting criteria, which limited direct cross-study comparability and made comprehensive stratification (e.g., by age, gender, treatment status, adherence, and Phe control) not feasible. Although plasma, serum and DBS represent distinct matrices collected and processed differently, we grouped them as blood-derived matrices due to the limited number of studies per matrix and the substantial variability in analytical methodologies and metabolite coverage across studies, consistent with the exploratory scope of this review. Stratification by age was not feasible due to the low number of studies analyzing pediatric and adult populations, and those who included both populations did not stratify their results by age. Importantly, most studies reported wide variability range in participants’ Phe levels, which limited the possibility of stratifying the results based on Phe concentration. Additionally, differences in metabolomic analytical methodologies and the sample collection time, further hindered the ability to stratify results across studies. Consequently, stratification was only possible based on the presence of dietary treatment for blood samples (Fig. 3). Notably, most metabolites were reported only once across studies, which limits the applicability of the observed findings for those metabolites (Supplementary Table 4). Moreover, pathway analyses should be interpreted with caution because only 235 out of 517 blood metabolites were assigned to pathways in MetaboAnalyst (Supplementary Table 4), and several pathways were represented by a limited number of assigned metabolites (Supplementary Tables 5 and 6). Lastly, only five urine-based studies were available, which limited comparison with the 23 blood-based studies (Fig. 3). These urine studies also presented both age and methodological differences, which further limited a robust comparative analysis. Overall, these findings underscore the urgent need for further metabolomic research and validation in larger cohorts.

## Conclusions

Overall, this work provides a novel and comprehensive systematic review that summarizes the significant differences in the metabolic profiles of patients with PKU, influenced by 544 metabolites, compared to healthy individuals across 26 studies. Notably, differences were mainly observed in blood samples (95%), with only 5% detected in urine samples, reflecting the limited use of this body fluid in only five studies. Therefore, further metabolomics research analyzing urine samples is encouraged for a more comprehensive understanding of the disease.

Our analysis identified extensive metabolic shifts in blood, where 60% of altered metabolites were upregulated and 40% were downregulated. While Phe-related markers dominated the upregulated group, we also observed significant involvement of lipids and amino acids, illustrating the complexity of the disease. Downregulated compounds were characterized by Trp metabolites (Trp and Kyn), among others. Notably, a subset of 35 metabolites (6% of the total) displayed inconsistent directions of change, likely due to clinical factors such as dietary adherence, metabolic control, nutrient supplementation, or pharmacological treatments, as well as the inherent differences between the blood matrices analyzed (DBS, plasma, and serum). The main limitations of this review stem from the high variability in patient characteristics, biological matrices, and analytical platforms, which limited robust stratification and the generalizability of some metabolite alterations.

This review offers valuable insights into the involvement of a broad range of metabolites and metabolic pathways, enhancing our understanding of the underlying biological mechanisms and pathophysiology of PKU. Monitoring these metabolic alterations in biological samples may support the optimization of disease management and therapeutic strategies. Future metabolomic studies incorporating multiple biological matrices and stratified analyses by demographic, clinical, and lifestyle factors are essential to fully characterize the metabolic phenotype of PKU and to refine dietary and overall management approaches.

## Supplementary Information

Below is the link to the electronic supplementary material.

Supplementary Material 1

Supplementary Material 2

## Data Availability

No datasets were generated or analysed during the current study.

## References

[CR1] Andrade, F., Cano, A., Unceta Suarez, M., Arza, A., Vinuesa, A., Ceberio, L., et al. (2021). Urine phenylacetylglutamine determination in patients with hyperphenylalaninemia. *Journal of Clinical Medicine,**10*(16), Article 3674. 10.3390/jcm10163674 34441968 10.3390/jcm10163674 PMC8396897

[CR2] Andrade, F., López-Suárez, O., Llarena, M., Couce, M. L., & Aldámiz-Echevarriá, L. (2017). Influence of phenylketonuria’s diet on dimethylated arginines and methylation cycle. *Medicine*. 10.1097/MD.0000000000007392 28682891 10.1097/MD.0000000000007392 PMC5502164

[CR3] Artuch, R., Colomé, C., Sierra, C., Brandi, N., Lambruschini, N., Campistol, J., et al. (2004). A longitudinal study of antioxidant status in phenylketonuric patients. *Clinical Biochemistry,**37*(3), 198–203. 10.1016/j.clinbiochem.2003.10.017 14972641 10.1016/j.clinbiochem.2003.10.017

[CR4] Blasco, H., Veyrat-Durebex, C., Bertrand, M., Patin, F., Labarthe, F., Henique, H., et al. (2017). A multiplatform metabolomics approach to characterize plasma levels of phenylalanine and tyrosine in phenylketonuria. *JIMD Reports,**32*, 69–79. 10.1007/8904_2016_568 27300702 10.1007/8904_2016_568 PMC5362559

[CR5] Blau, N., Van Spronsen, F. J., & Levy, H. L. (2010). Phenylketonuria. *Lancet,**376*(9750), 1417–1427. 10.1016/S0140-6736(10)60961-0 20971365 10.1016/S0140-6736(10)60961-0

[CR6] Bonte, R., Bongaerts, M., Demirdas, S., Langendonk, J. G., Huidekoper, H. H., Williams, M., et al. (2019). Untargeted metabolomics-based screening method for inborn errors of metabolism using semi-automatic sample preparation with an UHPLC-orbitrap-MS platform. *Metabolites*. 10.3390/metabo9120289 31779119 10.3390/metabo9120289 PMC6950026

[CR7] Boulet, L., Besson, G., van Noolen, L., Faure, P., ECOPHEN Study Group, Maillot, F., & Corne, C. (2020). Tryptophan metabolism in phenylketonuria: A French adult cohort study. *Journal of Inherited Metabolic Disease,**43*(5), 944–951. 10.1002/jimd.12250 32392388 10.1002/jimd.12250

[CR8] Cannet, C., Pilotto, A., Rocha, J. C., Schäfer, H., Spraul, M., Berg, D., et al. (2020). Lower plasma cholesterol, LDL-cholesterol and LDL-lipoprotein subclasses in adult phenylketonuria (PKU) patients compared to healthy controls: Results of NMR metabolomics investigation. *Orphanet Journal of Rare Diseases,**15*(1), Article 61. 10.1186/s13023-020-1329-5 32106880 10.1186/s13023-020-1329-5 PMC7047385

[CR9] Carrard, J., Guerini, C., Appenzeller-Herzog, C., Infanger, D., Königstein, K., Streese, L., et al. (2022). The metabolic signature of cardiorespiratory fitness: A systematic review. *Sports Medicine*. 10.1007/s40279-021-01590-y 34757595 10.1007/s40279-021-01590-y PMC8891196

[CR10] Coene, K. L. M., Kluijtmans, L. A. J., van der Heeft, E., Engelke, U. F. H., de Boer, S., Hoegen, B., et al. (2018). Next-generation metabolic screening: Targeted and untargeted metabolomics for the diagnosis of inborn errors of metabolism in individual patients. *Journal of Inherited Metabolic Disease,**41*(3), 337–353. 10.1007/s10545-017-0131-6 29453510 10.1007/s10545-017-0131-6 PMC5959972

[CR11] de Groot, M. J., Sijens, P. E., Reijngoud, D. J., Paans, A. M., & van Spronsen, F. J. (2015). Phenylketonuria: Brain phenylalanine concentrations relate inversely to cerebral protein synthesis. *Journal of Cerebral Blood Flow and Metabolism,**35*(2), 200–205. 10.1038/jcbfm.2014.183 25352046 10.1038/jcbfm.2014.183 PMC4426736

[CR12] Dobrowolski, S. F., Phua, Y. L., Vockley, J., Goetzman, E., & Blair, H. C. (2022). Phenylketonuria oxidative stress and energy dysregulation: Emerging pathophysiological elements provide interventional opportunity. *Molecular Genetics and Metabolism*. 10.1016/j.ymgme.2022.03.012 35379539 10.1016/j.ymgme.2022.03.012 PMC9832337

[CR13] Douglas, T. D., Jinnah, H. A., Bernhard, D., & Singh, R. H. (2013). The effects of sapropterin on urinary monoamine metabolites in phenylketonuria. *Molecular Genetics and Metabolism,**109*(3), 243–250. 10.1016/j.ymgme.2013.04.017 23712020 10.1016/j.ymgme.2013.04.017

[CR14] Drzymała-Czyż, S., Kałuzny, Ł, Krzyzanowska-Jankowska, P., Walkowiak, D., Mozrzymas, R., & Walkowiak, J. (2018). Deficiency of long-chain polyunsaturated fatty acids in phenylketonuria: A cross-sectional study. *Acta Biochimica Polonica,**65*(2), 303–308. 10.18388/abp.2018_2565 29913481 10.18388/abp.2018_2565

[CR15] Dybal, E., Maillot, F., Feillet, F., Fouilhoux, A., Astudillo, L., Lavigne, C., et al. (2025). Bone mineral density in French adults with early-treated phenylketonuria. *Molecular Genetics and Metabolism*. 10.1016/j.ymgme.2025.109044 39919675 10.1016/j.ymgme.2025.109044

[CR16] Firman, S. J., Ramachandran, R., & Whelan, K. (2022). Knowledge, perceptions and behaviours regarding dietary management of adults living with phenylketonuria. *Journal of Human Nutrition and Dietetics,**35*(6), 1016–1029. 10.1111/jhn.13015 35419899 10.1111/jhn.13015 PMC9790708

[CR17] Gassió, R., González, M. J., Sans, O., Artuch, R., Sierra, C., Ormazabal, A., et al. (2019). Prevalence of sleep disorders in early-treated phenylketonuric children and adolescents. Correlation with dopamine and serotonin status. *European Journal of Paediatric Neurology,**23*(5), 685–691. 10.1016/j.ejpn.2019.08.005 31522993 10.1016/j.ejpn.2019.08.005

[CR18] Guerra, I. M. S., Diogo, L., Pinho, M., Melo, T., Domingues, P., Domingues, M. R., & Moreira, A. S. P. (2021). Plasma phospholipidomic profile differs between children with phenylketonuria and healthy children. *Journal of Proteome Research,**20*(5), 2651–2661. 10.1021/acs.jproteome.0c01052 33819046 10.1021/acs.jproteome.0c01052

[CR19] Guerra, I. M. S., Ferreira, H. B., Neves, B., Melo, T., Diogo, L. M., Domingues, M. R., & Moreira, A. S. P. (2020). Lipids and phenylketonuria: Current evidences pointed the need for lipidomics studies. *Archives of Biochemistry and Biophysics*. 10.1016/j.abb.2020.108431 32461102 10.1016/j.abb.2020.108431

[CR20] Haijes, H. A., Willemsen, M., van der Ham, M., Gerrits, J., Pras-Raves, M. L., Prinsen, H. C. M. T., et al. (2019). Direct infusion-based metabolomics identifies metabolic disease in patients’ dried blood spots and plasma. *Metabolites*. 10.3390/metabo9010012 30641898 10.3390/metabo9010012 PMC6359237

[CR21] Hampe, M. H., Panaskar, S. N., Yadav, A. A., & Ingale, P. W. (2017). Gas chromatography/mass spectrometry-based urine metabolome study in children for inborn errors of metabolism: An Indian experience. *Clinical Biochemistry,**50*(3), 121–126. 10.1016/j.clinbiochem.2016.10.015 27784639 10.1016/j.clinbiochem.2016.10.015

[CR22] Hertz, L. (2013). The glutamate-glutamine (GABA) cycle: Importance of late postnatal development and potential reciprocal interactions between biosynthesis and degradation. *Frontiers in Endocrinology*. 10.3389/fendo.2013.00059 23750153 10.3389/fendo.2013.00059 PMC3664331

[CR23] Hillert, A., Anikster, Y., Belanger-Quintana, A., Burlina, A., Burton, B. K., Carducci, C., et al. (2020). The Genetic landscape and epidemiology of phenylketonuria. *American Journal of Human Genetics,**107*(2), 234–250. 10.1016/j.ajhg.2020.06.006 32668217 10.1016/j.ajhg.2020.06.006 PMC7413859

[CR24] Hoegen, B., Hampstead, J. E., Engelke, U. F. H., Kulkarni, P., Wevers, R. A., Brunner, H. G., et al. (2022). Application of metabolite set enrichment analysis on untargeted metabolomics data prioritises relevant pathways and detects novel biomarkers for inherited metabolic disorders. *Journal of Inherited Metabolic Disease,**45*(4), 682–695. 10.1002/jimd.12522 35546254 10.1002/jimd.12522 PMC9544878

[CR25] Hollak, C. E. M., & Lachmann, R. (Eds.). (2016). *Inherited Metabolic Disease in Adults: A Clinical Guide*. Oxford Monographs on Medical Genetics. online edn, Oxford Academic, 1 Sept. 201610.1093/med/9780199972135.001.0001

[CR26] Hou, X. W., Wang, Y., Ke, C., & Pan, C. W. (2023). Metabolomics facilitates the discovery of metabolic profiles and pathways for myopia: A systematic review. *Eye (London, England),**37*(4), 670–677. 10.1038/s41433-022-02019-0 35322213 10.1038/s41433-022-02019-0 PMC9998863

[CR27] Jacob, M., Malkawi, A., Albast, N., Al Bougha, S., Lopata, A., Dasouki, M., & Abdel Rahman, A. M. (2018). A targeted metabolomics approach for clinical diagnosis of inborn errors of metabolism. *Analytica Chimica Acta,**1025*, 141–153. 10.1016/j.aca.2018.03.058 29801603 10.1016/j.aca.2018.03.058

[CR28] Kanehisa, M., Sato, Y., Kawashima, M., Furumichi, M., & Tanabe, M. (2016). KEGG as a reference resource for gene and protein annotation. *Nucleic Acids Research*, *44*(D1), D457–D462. 10.1093/nar/gkv1070 26476454 10.1093/nar/gkv1070 PMC4702792

[CR29] Kong, S. W., & Hernandez-Ferrer, C. (2019). Assessment of coverage for endogenous metabolites and exogenous chemical compounds using an untargeted metabolomics platform. *Biocomputing*, 587–598. 10.1142/9789811215636_0052 PMC6910716 31797630

[CR30] Li, N., & Zhao, H. (2021). Role of carnitine in non-alcoholic fatty liver disease and other related diseases: An update. *Frontiers in Medicine*. 10.3389/fmed.2021.689042 34434943 10.3389/fmed.2021.689042 PMC8381051

[CR31] Liang, L., Ye, J., Han, L., Qiu, W., Zhang, H., Yu, Y., et al. (2020). Examining the blood amino acid status in pretherapeutic patients with hyperphenylalaninemia. *Journal of Clinical Laboratory Analysis,**34*(3), Article e23106. 10.1002/jcla.23106 31762087 10.1002/jcla.23106 PMC7083473

[CR32] Lumbreras, B., Porta, M., Márquez, S., Pollán, M., Parker, L. A., & Hernández-Aguado, I. (2008). QUADOMICS: An adaptation of the Quality Assessment of Diagnostic Accuracy Assessment (QUADAS) for the evaluation of the methodological quality of studies on the diagnostic accuracy of ’-omics’-based technologies. *Clinical Biochemistry*, *41*(16–17), 1316–1325. 10.1016/j.clinbiochem.2008.06.018 18652812 10.1016/j.clinbiochem.2008.06.018

[CR33] MacDonald, A., Gokmen-Ozel, H., Van Rijn, M., & Burgard, P. (2010, December). The reality of dietary compliance in the management of phenylketonuria. *Journal of Inherited Metabolic Disease*. 10.1007/s10545-010-9073-y 10.1007/s10545-010-9073-y 20373144

[CR34] MacDonald, A., Van Wegberg, A. M. J., Ahring, K., Beblo, S., Bélanger-Quintana, A., & Burlina, A. (2020). PKU dietary handbook to accompany PKU guidelines. *Orphanet Journal of Rare Diseases*. 10.1186/s13023-020-01391-y 32605583 10.1186/s13023-020-01391-y PMC7329487

[CR35] Miller, M. J., Kennedy, A. D., Eckhart, A. D., Burrage, L. C., Wulff, J. E., Miller, L. A. D., et al. (2015). Untargeted metabolomic analysis for the clinical screening of inborn errors of metabolism. *Journal of Inherited Metabolic Disease,**38*(6), 1029–1039. 10.1007/s10545-015-9843-7 25875217 10.1007/s10545-015-9843-7 PMC4626538

[CR36] Mordaunt, D., Cox, D., & Fuller, M. (2020). Metabolomics to improve the diagnostic efficiency of inborn errors of metabolism. *International Journal of Molecular Sciences*. 10.3390/ijms21041195 32054038 10.3390/ijms21041195 PMC7072749

[CR37] Moritz, L., Klotz, K., Grünert, S. C., Hannibal, L., & Spiekerkoetter, U. (2023). Metabolic phenotyping in phenylketonuria reveals disease clustering independently of metabolic control. *Molecular Genetics and Metabolism,**138*(3), Article 107509. 10.1016/j.ymgme.2023.107509 36791482 10.1016/j.ymgme.2023.107509

[CR38] Mütze, U., Beblo, S., Kortz, L., Matthies, C., Koletzko, B., Bruegel, M., et al. (2012). Metabolomics of dietary fatty acid restriction in patients with phenylketonuria. *PLoS ONE,**7*(8), Article e43021. 10.1371/journal.pone.0043021 22912778 10.1371/journal.pone.0043021 PMC3418234

[CR39] Page, M. J., Moher, D., Bossuyt, P. M., et al. (2021). PRISMA 2020 explanation and elaboration: Updated guidance and exemplars for reporting systematic reviews. *Bmj*, *372*, n160. 10.1136/bmj.n160(n.d.). 33781993 10.1136/bmj.n160 PMC8005925

[CR40] Pan, Z., Gu, H., Talaty, N., Chen, H., Shanaiah, N., Hainline, B. E., et al. (2007). Principal component analysis of urine metabolites detected by NMR and DESI-MS in patients with inborn errors of metabolism. *Analytical and Bioanalytical Chemistry,**387*(2), 539–549. 10.1007/s00216-006-0546-7 16821030 10.1007/s00216-006-0546-7

[CR41] Pang, Z., Lu, Y., Zhou, G., Hui, F., Xu, L., Viau, C., et al. (2024). MetaboAnalyst 6.0: Towards a unified platform for metabolomics data processing, analysis and interpretation. *Nucleic Acids Research,**52*(W1), W398–W406. 10.1093/nar/gkae253 38587201 10.1093/nar/gkae253 PMC11223798

[CR42] Parker, L. A., Saez, N. G., Lumbreras, B., Porta, M., & Hernández-Aguado, I. (2010). Methodological deficits in diagnostic research using “-Omics” technologies: Evaluation of the QUADOMICS tool and quality of recently published studies. *PLoS ONE,**5*(7), Article e11419. 10.1371/journal.pone.0011419 20625481 10.1371/journal.pone.0011419 PMC2896422

[CR43] Perry, T. L., Hansen, S., Tischler, B., Bunting, R., & Diamond, S. (1970). Glutamine depletion in phenylketonuria. *The New England Journal of Medicine,**282*(14), 761–766. 10.1056/NEJM197004022821401 5416968 10.1056/NEJM197004022821401

[CR44] Rausell, D., García-Blanco, A., Correcher, P., Vitoria, I., Vento, M., & Cháfer-Pericás, C. (2019). Newly validated biomarkers of brain damage may shed light into the role of oxidative stress in the pathophysiology of neurocognitive impairment in dietary restricted phenylketonuria patients. *Pediatric Research*. 10.1038/s41390-018-0202-x 30333522 10.1038/s41390-018-0202-x

[CR45] Ribas, G. S., Sitta, A., Wajner, M., & Vargas, C. R. (2011). Oxidative stress in phenylketonuria: What is the evidence? *Cellular and Molecular Neurobiology,**31*(5), 653–662. 10.1007/s10571-011-9693-2 21516352 10.1007/s10571-011-9693-2 PMC11498541

[CR46] Rondanelli, M., Porta, F., Gasparri, C., Barrile, G. C., Cavioni, A., Mansueto, F., et al. (2023). A food pyramid for adult patients with phenylketonuria and a systematic review on the current evidences regarding the optimal dietary treatment of adult patients with PKU. *Clinical Nutrition,**42*(5), 732–763. 10.1016/j.clnu.2023.03.007 37001196 10.1016/j.clnu.2023.03.007

[CR47] Sanayama, Y., Nagasaka, H., Takayanagi, M., Ohura, T., Sakamoto, O., Ito, T., et al. (2011). Experimental evidence that phenylalanine is strongly associated to oxidative stress in adolescents and adults with phenylketonuria. *Molecular Genetics and Metabolism,**103*(3), 220–225. 10.1016/j.ymgme.2011.03.019 21514861 10.1016/j.ymgme.2011.03.019

[CR48] Sanjurjo, P., Perteagudo, L., Soriano, J. R., Vilaseca, A., & Campistol, J. (1994). Polyunsaturated fatty acid status in patients with phenylketonuria. *Journal of Inherited Metabolic Disease,**17*(6), 704–709. 7707693 10.1007/BF00712012

[CR49] Schoen, M. S., & Singh, R. H. (2022). Plasma metabolomic profile changes in females with phenylketonuria following a camp intervention. *American Journal of Clinical Nutrition,**115*(3), 811–821. 10.1093/ajcn/nqab400 34864852 10.1093/ajcn/nqab400 PMC8895208

[CR50] Schoen, M. S., Ramakrishnan, U., Alvarez, J. A., Ziegler, T. R., Cui, X., & Singh, R. H. (2022). Characterization of choline nutriture among adults and children with phenylketonuria. *Nutrients,**14*(19), Article 4056. 10.3390/nu14194056 36235708 10.3390/nu14194056 PMC9572308

[CR51] Schousboe, A., Scafidi, S., Bak, L. K., Waagepetersen, H. S., & McKenna, M. C. (2014). Glutamate metabolism in the brain focusing on astrocytes. *Advances in Neurobiology*. 10.1007/978-3-319-08894-5_2 25236722 10.1007/978-3-319-08894-5_2 PMC4667713

[CR52] Schulpis, K. H., Karikas, G. A., Tjamouranis, J., Michelakakis, H., & Tsakiris, S. (2002). Acetylcholinesterase activity and biogenic amines in phenylketonuria. *Clinical Chemistry,**48*(10), 1794–1796. 10.1093/CLINCHEM/48.10.1794 12324501

[CR53] Sirtori, L. R., Dutra-Filho, C. S., Fitarelli, D., Sitta, A., Haeser, A., Barschak, A. G., et al. (2005). Oxidative stress in patients with phenylketonuria. *Biochimica et Biophysica Acta - Molecular Basis of Disease,**1740*(1), 68–73. 10.1016/j.bbadis.2005.02.005 10.1016/j.bbadis.2005.02.005 15878743

[CR54] Sitta, A., Barschak, A. G., Deon, M., De Mari, J. F., Barden, A. T., Vanzin, C. S., et al. (2009). L-carnitine blood levels and oxidative stress in treated phenylketonuric patients. *Cellular and Molecular Neurobiology,**29*(2), 211–218. 10.1007/s10571-008-9313-y 18814025 10.1007/s10571-008-9313-y PMC11506149

[CR55] Stroup, B. M., Nair, N., Murali, S. G., Broniowska, K., Rohr, F., Levy, H. L., & Ney, D. M. (2018). Metabolomic markers of essential fatty acids, carnitine, and cholesterol metabolism in adults and adolescents with phenylketonuria. *The Journal of Nutrition,**148*(2), 194–201. 10.1093/jn/nxx039 29490096 10.1093/jn/nxx039 PMC6251508

[CR56] Ulaszewska, M. M., Weinert, C. H., Trimigno, A., Portmann, R., Andres Lacueva, C., & Badertscher, R. (2019). Nutrimetabolomics: An integrative action for metabolomic analyses in human nutritional studies. *Molecular Nutrition & Food Research*. 10.1002/mnfr.201800384 10.1002/mnfr.201800384 30176196

[CR57] Václavík, J., Coene, K. L. M., Vrobel, I., Najdekr, L., Friedecký, D., Karlíková, R., et al. (2018). Structural elucidation of novel biomarkers of known metabolic disorders based on multistage fragmentation mass spectra. *Journal of Inherited Metabolic Disease,**41*(3), 407–414. 10.1007/s10545-017-0109-4 29139026 10.1007/s10545-017-0109-4

[CR58] van Spronsen, F. J., Blau, N., Harding, C., Burlina, A., Longo, N., & Bosch, A. M. (2021). Phenylketonuria. *Nature Reviews Disease Primers*. 10.1038/s41572-021-00267-0 34017006 10.1038/s41572-021-00267-0 PMC8591558

[CR59] van Wegberg, A. M. J., MacDonald, A., Ahring, K., Bélanger-Quintana, A., Beblo, S., & Blau, N. (2025). European guidelines on diagnosis and treatment of phenylketonuria: First revision. *Molecular Genetics and Metabolism*. 10.1016/j.ymgme.2025.109125 40378670 10.1016/j.ymgme.2025.109125

[CR60] van Wegberg, A. M. J., MacDonald, A., Ahring, K., Bélanger-Quintana, A., Blau, N., & Bosch, A. M. (2017). The complete European guidelines on phenylketonuria: Diagnosis and treatment. *Orphanet Journal of Rare Diseases*. 10.1186/s13023-017-0685-2 29025426 10.1186/s13023-017-0685-2 PMC5639803

[CR61] Vilaseca, M. A., Briones, P., Ferrer, I., Campistol, J., Riverola, A., Castillo, P., & Ramon, F. (1993). Controlled diet in phenylketonuria may cause serum carnitine deficiency. *Journal of Inherited Metabolic Disease,**16*(1), 101–104. 10.1007/BF00711322 8487489 10.1007/BF00711322

[CR62] Vilaseca, M. A., Lambruschini, N., Gómez-López, L., Gutiérrez, A., Moreno, J., Tondo, M., et al. (2010). Long-chain polyunsaturated fatty acid status in phenylketonuric patients treated with tetrahydrobiopterin. *Clinical Biochemistry,**43*(4–5), 411–415. 10.1016/j.clinbiochem.2009.11.013 19948162 10.1016/j.clinbiochem.2009.11.013

[CR63] Wan, Z., Rosenbaum, E. R., Liu, W., Song, B., Yue, X., Kong, Y., et al. (2022). Benchmark examination of blood amino acids patterns in phenylketonuria neonates and young children on phenylalanine-restricted dietary treatment. *Fetal and Pediatric Pathology,**41*(3), 443–450. 10.1080/15513815.2020.1846647 33198547 10.1080/15513815.2020.1846647

[CR64] Weigel, C., Kiener, C., Meier, N., Schmid, P., Rauh, M., Rascher, W., & Knerr, I. (2008). Carnitine status in early-treated children, adolescents and young adults with phenylketonuria on low phenylalanine diets. *Annals of Nutrition and Metabolism*, *53*(2), 91–95. 10.1159/000165356 18946205 10.1159/000165356

[CR65] Whiting, P., Rutjes, A. W. S., Reitsma, J. B., Bossuyt, P. M. M., & Kleijnen, J. (2003). The development of QUADAS: A tool for the quality assessment of studies of diagnostic accuracy included in systematic reviews. *BMC Medical Research Methodology,**3*, Article 25. 10.1186/1471-2288-3-25 14606960 10.1186/1471-2288-3-25 PMC305345

[CR66] Wild, J., Shanmuganathan, M., Hayashi, M., Potter, M., & Britz-Mckibbin, P. (2019). Metabolomics for improved treatment monitoring of phenylketonuria: Urinary biomarkers for non-invasive assessment of dietary adherence and nutritional deficiencies. *Analyst,**144*(22), 6595–6608. 10.1039/c9an01642b 31608347 10.1039/c9an01642b

[CR67] Wishart, D. S., Guo, A. C., Oler, E., Wang, F., Anjum, A., Peters, H., et al. (2022). HMDB 5.0: The Human Metabolome Database for 2022. *Nucleic Acids Research,**50*(D1), D622–D631. 10.1093/nar/gkab1062 34986597 10.1093/nar/gkab1062 PMC8728138

[CR68] Xiong, X., Sheng, X., Liu, D., Zeng, T., Peng, Y., & Wang, Y. (2015). A GC/MS-based metabolomic approach for reliable diagnosis of phenylketonuria. *Analytical and Bioanalytical Chemistry*, *407*(29), 8825–8833. 10.1007/s00216-015-9041-3 26410738 10.1007/s00216-015-9041-3

