# Supplementary material for: Metabolomic profiling in phenylketonuria: a systematic review of human studies
Source: Metabolomics. 2026 Mar 7;22(2):39. doi: 10.1007/s11306-026-02416-6 (PMC12967636; doi:10.1007/s11306-026-02416-6)
Supplement: Supplementary file 1 — Supplementary Material 1 [file 11306_2026_2416_MOESM1_ESM.docx]

**SUPPLEMENTARY MATERIAL**

**Metabolomic Profiling in Phenylketonuria: A Systematic Review of Human Studies**

Arnau Gonzalez-Rodriguez^1,2^, Mireia Urpi-Sarda^1,2,3^*, Blanca Barrau-Martinez^1,2^, Francesc M Campins-Machado^1,2^, Hadia Bakkali-Aissaoui^1^, Adriana Pané^4,5^, Pedro J Moreno^6,7,8^, Emilio Ortega^4,5^, Judit Garcia-Villoria^8,9^, Aida Ormazabal^8,10^, Dolores Garcia-Arenas^1,11^, Carme Junqué^12^, Gloria Garrabou^6,7,8^, Rafael Llorach^1,2,3^*, on behalf of the Consortium PKU.cat

1: Departament de Nutrició, Ciències de l’Alimentació i Gastronomia, Facultat de Farmàcia i Ciències de l’Alimentació, Campus de l’Alimentació de Torribera, Universitat de Barcelona (UB), 08921, Santa Coloma de Gramenet, Spain.

2: Institut de Recerca en Nutrició i Seguretat Alimentària (INSA-UB), Campus de l’Alimentació de Torribera, Universitat de Barcelona (UB), 08921, Santa Coloma de Gramenet, Spain.

3: Centro de Investigación Biomédica en Red de Fragilidad y Envejecimiento Saludable (CIBERFES), Instituto de Salud Carlos III (ISCIII), 28029, Madrid, Spain.

4: Endocrinology and Nutrition Department, Adult Inherited Metabolic Disorders Unit (UECMA), Hospital Clínic de Barcelona, 08036, Barcelona, Spain.

5: Centro de Investigación Biomédica en Red de Fisiopatología de la Obesidad y Nutrición (CIBEROBN), Instituto de Salud Carlos III (ISCIII), 28029, Madrid, Spain.

6: Internal Medicine Department, Adult Inherited Metabolic Disorders Unit (UECMA), Hospital Clínic de Barcelona, 08036, Barcelona, Spain.

7: Inherited Metabolic Diseases and Muscle Disorders Research Laboratory, Centre de Recerca Biomèdica CELLEX - Institut d'Investigacions Biomèdiques August Pi i Sunyer (IDIBAPS) and Faculty of Medicine and Health Sciences, University of Barcelona, 08036, Barcelona, Spain.

8: Centro de Investigación Biomédica en Red de Enfermedades Raras (CIBERER), Instituto de Salud Carlos III (ISCIII), 28029, Madrid, Spain.

9: Section of Inborn Errors of Metabolism-IBC, Biochemistry and Molecular Genetics Department, Hospital Clínic de Barcelona, IDIBAPS, 08036, Barcelona, Spain.

10: Clinical Biochemistry Department, Sant Joan de Déu Hospital, Institut de Recerca Sant Joan de Déu, Esplugues de Llobregat, 08950, Barcelona, Spain.

11: Inborn Errors of Metabolism Unit, Sant Joan de Déu Hospital, Esplugues de Llobregat, 08950, Barcelona, Spain.

12: Fundació de Recerca Clínic Barcelona-Institut d'Investigacions Biomèdiques August Pi I Sunyer (FRCB-IDIBAPS), Institute of Neurosciences, Department of Medicine, University of Barcelona, Centro de Investigación Biomédica en Red sobre Enfermedades Neurodegenerativas (CIBERNED), 08036, Barcelona, Spain.

**Correspondence**

***Corresponding authors:** **Dr. Mireia Urpi-Sarda**, E-mail: [murpi@ub.edu](mailto:murpi@ub.edu). <https://orcid.org/0000-0002-4064-5175>. **Dr. Rafael Llorach,** E-mail: [rafallorach@ub.edu](mailto:rafallorach@ub.edu). <https://orcid.org/0000-0002-5215-4445>

**SUPPLEMENTARY MATERIAL**

**Supplementary Table S1.** Syntax used in the three databases for the systematic literature search.

**Supplementary Table S2.** Items of QUADOMICS tool used in this systematic review

**Supplementary Table S3.** Quality assessment of the selected studies using QUADOMICS tool.

**Supplementary Table S4.** Up- and downregulated metabolites in blood and urine and their pathways.

**Supplementary Table S5.** Summary of the Pathway Analysis of upregulated blood metabolites in PKU.

**Supplementary Table S6.** Summary of the Pathway Analysis of downregulated blood metabolites in PKU.

**Supplementary Table S1.** Syntax used in the three databases for the systematic literature search.

| **DATABASE** | **SYNTAX** |
| --- | --- |
| **PubMed®** | (("human*"[Title/Abstract] OR "subject*"[Title/Abstract] OR "adult*"[Title/Abstract] OR "child*"[Title/Abstract] OR "men"[Title/Abstract] OR "male"[Title/Abstract] OR "women"[Title/Abstract] OR "female"[Title/Abstract] OR "patient*"[Title/Abstract] OR "volunteer*"[Title/Abstract] OR "participant*"[Title/Abstract] OR "adolescent*"[Title/Abstract] OR "overweight*"[Title/Abstract] OR "infant*"[Title/Abstract] OR "newborn*"[Title/Abstract] OR "neonate*"[Title/Abstract]) AND ("metabonomic*"[Title/Abstract] OR "metabolic profile"[Title/Abstract] OR "metabolomic profile"[Title/Abstract] OR "biomarker*"[Title/Abstract] OR "metabol*"[Title/Abstract] OR "fingerprint*"[Title/Abstract]) AND ("PKU"[Title/Abstract] OR "phenylketonuri*"[Title/Abstract] OR "Hyperphenylalaninemi*"[Title/Abstract] OR "PAH"[Title/Abstract] OR "BH4"[Title/Abstract] OR "Tetrahydrobiopterin"[Title/Abstract] OR "DHPR"[Title/Abstract] OR "Dihydropteridine Reductase"[Title/Abstract] OR "PTPS"[Title/Abstract] OR "GTP cyclohydrolase"[Title/Abstract] OR "Pterin-4-alpha-carbinolamine dehydratase"[Title/Abstract] OR "PCBD1"[Title/Abstract] OR "Sepiapterin reductase"[Title/Abstract]) AND ("serum"[Title/Abstract] OR "urine*"[Title/Abstract] OR "plasma"[Title/Abstract] OR "feces"[Title/Abstract] OR "faeces"[Title/Abstract] OR "blood"[Title/Abstract] OR "saliva"[Title/Abstract]) AND ("LC-MS"[Title/Abstract] OR "LC/MS"[Title/Abstract] OR "GC-MS"[Title/Abstract] OR "GC/MS"[Title/Abstract] OR "NMR"[Title/Abstract] OR "chromatography"[Title/Abstract] OR "nuclear magnetic resonance"[Title/Abstract] OR "gas chromatography"[Title/Abstract] OR "mass spectrometry"[Title/Abstract])) |
| **Scopus®** | TITLE-ABS-KEY ( "human*"  OR  "subject*"  OR  "adult*"  OR  "child*"  OR  "men"  OR  "male"  OR  "women"  OR  "female"  OR  "patient*"  OR  "volunteer*"  OR  "participant*"  OR  "adolescent*"  OR  "overweight*"  OR  "infant*"  OR  "newborn*"  OR  "neonate*" )  AND  TITLE-ABS-KEY ( "metabonomic*"  OR  "metabolic profile"  OR  "metabolomic profile"  OR  "biomarker*"  OR  "metabol*"  OR  "fingerprint*" )  AND  TITLE-ABS-KEY ( "PKU"  OR  "phenylketonuri*"  OR  "Hyperphenylalaninemi*"  OR  "PAH"  OR  "BH4"  OR  "Tetrahydrobiopterin"  OR  "DHPR"  OR  "Dihydropteridine Reductase"  OR  "PTPS"  OR  "GTP cyclohydrolase"  OR  "Pterin-4-alpha-carbinolamine dehydratase"  OR  "PCBD1"  OR  "Sepiapterin reductase" )  AND  TITLE-ABS-KEY ( "serum"  OR  "urine*"  OR  "plasma"  OR  "feces"  OR  "faeces"  OR  "blood"  OR  "saliva" )  AND  TITLE-ABS-KEY ( "LC-MS"  OR  "LC/MS"  OR  "GC-MS"  OR  "GC/MS"  OR  "NMR"  OR  "chromatography"  OR  "nuclear magnetic resonance"  OR  "gas chromatography"  OR  "mass spectrometry") |
| **Web of Science™** | (TI=(("human*" OR "subject*" OR "adult*" OR "child*" OR "men" OR "male" OR "women" OR "female" OR "patient*" OR "volunteer*" OR "participant*" OR "adolescent*" OR "overweight*" OR "infant*" OR "newborn*" OR "neonate*") AND ("metabonomic*" OR "metabolic profile" OR "metabolomic profile" OR "biomarker*" OR "metabol*" OR "fingerprint*") AND ("PKU" OR "phenylketonuri*" OR "Hyperphenylalaninemi*" OR "PAH" OR "BH4" OR "Tetrahydrobiopterin" OR "DHPR" OR "Dihydropteridine Reductase" OR "PTPS" OR "GTP cyclohydrolase" OR "Pterin-4-alpha-carbinolamine dehydratase" OR "PCBD1" OR "Sepiapterin reductase") AND ("serum" OR "urine*" OR "plasma" OR "feces" OR "faeces" OR "blood" OR "saliva") AND ("LC-MS" OR "LC/MS" OR "GC-MS" OR "GC/MS" OR "NMR" OR "chromatography" OR "nuclear magnetic resonance" OR "gas chromatography" OR "mass spectrometry"))) OR AB=(("human*" OR "subject*" OR "adult*" OR "child*" OR "men" OR "male" OR "women" OR "female" OR "patient*" OR "volunteer*" OR "participant*" OR "adolescent*" OR "overweight*" OR "infant*" OR "newborn*" OR "neonate*") AND ("metabonomic*" OR "metabolic profile" OR "metabolomic profile" OR "biomarker*" OR "metabol*" OR "fingerprint*") AND ("PKU" OR "phenylketonuri*" OR "Hyperphenylalaninemi*" OR "PAH" OR "BH4" OR "Tetrahydrobiopterin" OR "DHPR" OR "Dihydropteridine Reductase" OR "PTPS" OR "GTP cyclohydrolase" OR "Pterin-4-alpha-carbinolamine dehydratase" OR "PCBD1" OR "Sepiapterin reductase") AND ("serum" OR "urine*" OR "plasma" OR "feces" OR "faeces" OR "blood" OR "saliva") AND ("LC-MS" OR "LC/MS" OR "GC-MS" OR "GC/MS" OR "NMR" OR "chromatography" OR "nuclear magnetic resonance" OR "gas chromatography" OR "mass spectrometry")) |

**Supplementary Table S2.** Items of QUADOMICS tool used in this systematic review (Carrard et al. 2022; Lumbreras et al. 2008).

| **Item** | **Description** |
| --- | --- |
| ***1*** | Were selection criteria clearly described? |
| ***3*** | Was the type of sample fully described? |
| ***4*** | Were the procedures and timing of biological sample collection with respect to clinical factors described with enough detail? |
| ***5*** | Were handling and pre-analytical procedures reported in sufficient detail and similar for the whole sample? And, if differences in procedures were reported, was their effect on the results assessed? |
| ***10*** | Was the execution of the index test described in sufficient detail to permit replication of the test? |
| ***11*** | Was the execution of the reference standard described in sufficient detail to permit its replication? |
| ***15*** | Were uninterpretable / intermediate test results reported? |
| ***16*** | Is it likely that the presence of over-fitting was avoided? |

**Supplementary Table S3.** Quality assessment of the selected studies using QUADOMICS tool (Carrard et al. 2022; Hou et al. 2023; Lumbreras et al. 2008).

| ***Article*** | ***1*** | ***3*** | ***4*** | ***5*** | ***10*** | ***11*** | ***15*** | ***Score*** |
| --- | --- | --- | --- | --- | --- | --- | --- | --- |
| Andrade et al., (2017) |  |  |  |  |  |  |  | 100% |
| Blasco et al., (2017) |  |  |  |  |  |  |  | 93% |
| Bonte et al., (2019) |  |  |  |  |  |  |  | 86% |
| Boulet et al., (2020) |  |  |  |  |  |  |  | 100% |
| Cannet et al., (2020) |  |  |  |  |  |  |  | 100% |
| Coene et al., (2018) |  |  |  |  |  |  |  | 86% |
| Douglas et al., (2013) |  |  |  |  |  |  |  | 93% |
| Drzymała-Czyż et al., (2018) |  |  |  |  |  |  |  | 100% |
| Guerra et al., (2021) |  |  |  |  |  |  |  | 100% |
| Haijes et al., (2019) |  |  |  |  |  |  |  | 71% |
| Hampe et al., (2017) |  |  |  |  |  |  |  | 71% |
| Hoegen et al., (2022) |  |  |  |  |  |  |  | 86% |
| Jacob et al., (2018) |  |  |  |  |  |  |  | 79% |
| Kong and Hernandez-Ferrer (2019) |  |  |  |  |  |  |  | 71% |
| Liang et al., (2020) |  |  |  |  |  |  |  | 100% |
| Miller et al., (2015) |  |  |  |  |  |  |  | 79% |
| Moritz et al., (2023) |  |  |  |  |  |  |  | 86% |
| Mütze et al., (2012) |  |  |  |  |  |  |  | 93% |
| Pan et al., (2007) |  |  |  |  |  |  |  | 71% |
| Schoen and Singh (2022) |  |  |  |  |  |  |  | 93% |
| Schulpis et al., (2002) |  |  |  |  |  |  |  | 79% |
| Stroup et al., (2018) |  |  |  |  |  |  |  | 93% |
| Václavík et al., (2018) |  |  |  |  |  |  |  | 71% |
| Wan et al., (2022) |  |  |  |  |  |  |  | 93% |
| Weigel et al., (2008) |  |  |  |  |  |  |  | 79% |
| Xiong et al., (2015) |  |  |  |  |  |  |  | 79% |

Green: 1 point; Yellow: 0.5 points; Red: 0 points.

**Supplementary Table S4.** Up- and downregulated metabolites in blood and urine and their pathways. See Excel File.

**Supplementary Table S5.** Summary of the Pathway Analysis of upregulated blood metabolites in patients with PKU.

| **Pathway Name** | **Match Status** | **p** | **-log(p)** | **Holm p** | **FDR** | **Impact** |
| --- | --- | --- | --- | --- | --- | --- |
| Arginine biosynthesis | 7/14 | 5.72E-07 | 6.24 | 4.57E-05 | 4.57E-05 | 0.51 |
| Phenylalanine metabolism | 5/8 | 6.71E-06 | 5.17 | 5.30E-04 | 2.68E-04 | 0.62 |
| Biosynthesis of unsaturated fatty acids | 8/36 | 9.48E-05 | 4.02 | 7.39E-03 | 1.94E-03 | 0 |
| Glutathione metabolism | 7/28 | 1.21E-04 | 3.92 | 9.33E-03 | 1.94E-03 | 0.43 |
| Alanine, aspartate and glutamate metabolism | 7/28 | 1.21E-04 | 3.92 | 9.33E-03 | 1.94E-03 | 0.67 |
| Phenylalanine, tyrosine and tryptophan biosynthesis | 3/4 | 3.03E-04 | 3.52 | 0.02 | 4.03E-03 | 1 |
| One carbon pool by folate | 6/26 | 6.17E-04 | 3.21 | 0.05 | 7.05E-03 | 0.24 |
| Butanoate metabolism | 4/15 | 3.07E-03 | 2.51 | 0.22 | 0.03 | 0 |
| Arginine and proline metabolism | 6/36 | 3.70E-03 | 2.43 | 0.27 | 0.03 | 0.38 |
| Taurine and hypotaurine metabolism | 3/8 | 3.73E-03 | 2.43 | 0.27 | 0.03 | 0.83 |
| Histidine metabolism | 4/16 | 3.97E-03 | 2.40 | 0.28 | 0.03 | 0.22 |

Notes. The term Impact refers to the pathway impact value calculated through the topology analysis by MetaboAnalyst. FDR, false discovery rate; PKU, phenylketonuria.

**Supplementary Table S6.** Summary of the Pathway Analysis of downregulated blood metabolites in patients with PKU.

| **Pathway Name** | **Match Status** | **p** | **-log(p)** | **Holm p** | **FDR** | **Impact** |
| --- | --- | --- | --- | --- | --- | --- |
| Valine, leucine and isoleucine biosynthesis | 6/8 | 8.79E-08 | 7.06 | 7.04E-06 | 7.04E-06 | 0 |
| Arginine and proline metabolism | 9/36 | 6.20E-06 | 5.21 | 4.90E-04 | 1.44E-04 | 0.40 |
| Glycerophospholipid metabolism | 9/36 | 6.20E-06 | 5.21 | 4.90E-04 | 1.44E-04 | 0.59 |
| Alanine, aspartate and glutamate metabolism | 8/28 | 7.22E-06 | 5.14 | 5.56E-04 | 1.44E-04 | 0.53 |
| Arginine biosynthesis | 5/14 | 1.36E-04 | 3.87 | 0.01 | 2.17E-03 | 0.27 |
| Histidine metabolism | 4/16 | 3.01E-03 | 2.52 | 0.23 | 0.04 | 0.22 |

Notes. The term Impact refers to the pathway impact value calculated through the topology analysis by MetaboAnalyst. FDR, false discovery rate; PKU, phenylketonuria.

**REFERENCES**

Andrade, F., López-Suárez, O., Llarena, M., Couce, M. L., & Aldámiz-Echevarriá, L. (2017). Influence of phenylketonuria’s diet on dimethylated arginines and methylation cycle. Medicine (United States), 96(27). https://doi.org/10.1097/MD.0000000000007392

Blasco, H., Veyrat-Durebex, C., Bertrand, M., Patin, F., Labarthe, F., Henique, H., et al. (2017). A multiplatform metabolomics approach to characterize plasma levels of phenylalanine and tyrosine in phenylketonuria. JIMD Reports, 32, 69–79. https://doi.org/10.1007/8904_2016_568

Bonte, R., Bongaerts, M., Demirdas, S., Langendonk, J. G., Huidekoper, H. H., Williams, M., et al. (2019). Untargeted metabolomics-based screening method for inborn errors of metabolism using semi-automatic sample preparation with an UHPLC-orbitrap-MS platform. Metabolites, 9(12). https://doi.org/10.3390/metabo9120289

Boulet, L., Besson, G., van Noolen, L., Faure, P., ECOPHEN Study Group, Maillot, F., & Corne, C. (2020). Tryptophan metabolism in phenylketonuria: A French adult cohort study. Journal of Inherited Metabolic Disease, 43(5), 944–951. https://doi.org/10.1002/jimd.12250

Cannet, C., Pilotto, A., Rocha, J. C., Schäfer, H., Spraul, M., Berg, D., et al. (2020). Lower plasma cholesterol, LDL-cholesterol and LDL-lipoprotein subclasses in adult phenylketonuria (PKU) patients compared to healthy controls: Results of NMR metabolomics investigation. Orphanet Journal of Rare Diseases, 15(1), 61. https://doi.org/10.1186/s13023-020-1329-5

Carrard, J., Guerini, C., Appenzeller-Herzog, C., Infanger, D., Königstein, K., Streese, L., et al. (2022, March 1). The Metabolic Signature of Cardiorespiratory Fitness: A Systematic Review. Sports Med. Springer Science and Business Media Deutschland GmbH. https://doi.org/10.1007/s40279-021-01590-y

Coene, K. L. M., Kluijtmans, L. A. J., van der Heeft, E., Engelke, U. F. H., de Boer, S., Hoegen, B., et al. (2018). Next-generation metabolic screening: targeted and untargeted metabolomics for the diagnosis of inborn errors of metabolism in individual patients. Journal of Inherited Metabolic Disease, 41(3), 337–353. https://doi.org/10.1007/s10545-017-0131-6

Douglas, T. D., Jinnah, H. A., Bernhard, D., & Singh, R. H. (2013). The effects of sapropterin on urinary monoamine metabolites in phenylketonuria. Molecular Genetics and Metabolism, 109(3), 243–250. https://doi.org/10.1016/j.ymgme.2013.04.017

Drzymała-Czyz, S., Kałuzny, Ł., Krzyzanowska-Jankowska, P., Walkowiak, D., Mozrzymas, R., & Walkowiak, J. (2018). Deficiency of long-chain polyunsaturated fatty acids in phenylketonuria: A cross-sectional study. Acta Biochimica Polonica, 65(2), 303–308. https://doi.org/10.18388/abp.2018_2565

Guerra, I. M. S., Diogo, L., Pinho, M., Melo, T., Domingues, P., Domingues, M. R., & Moreira, A. S. P. (2021). Plasma Phospholipidomic Profile Differs between Children with Phenylketonuria and Healthy Children. Journal of Proteome Research, 20(5), 2651–2661. https://doi.org/10.1021/acs.jproteome.0c01052

Haijes, H. A., Willemsen, M., van der Ham, M., Gerrits, J., Pras-Raves, M. L., Prinsen, H. C. M. T., et al. (2019). Direct infusion based metabolomics identifies metabolic disease in patients’ dried blood spots and plasma. Metabolites, 9(1). https://doi.org/10.3390/metabo9010012

Hampe, M. H., Panaskar, S. N., Yadav, A. A., & Ingale, P. W. (2017). Gas chromatography/mass spectrometry-based urine metabolome study in children for inborn errors of metabolism: An Indian experience. Clinical Biochemistry, 50(3), 121–126. https://doi.org/10.1016/j.clinbiochem.2016.10.015

Hoegen, B., Hampstead, J. E., Engelke, U. F. H., Kulkarni, P., Wevers, R. A., Brunner, H. G., et al. (2022). Application of metabolite set enrichment analysis on untargeted metabolomics data prioritises relevant pathways and detects novel biomarkers for inherited metabolic disorders. Journal of Inherited Metabolic Disease, 45(4), 682–695. https://doi.org/10.1002/jimd.12522

Hou, X. W., Wang, Y., Ke, C., & Pan, C. W. (2023). Metabolomics facilitates the discovery of metabolic profiles and pathways for myopia: A systematic review. Eye (Lond), 37(4), 670–677. https://doi.org/10.1038/s41433-022-02019-0

Jacob, M., Malkawi, A., Albast, N., Al Bougha, S., Lopata, A., Dasouki, M., & Abdel Rahman, A. M. (2018). A targeted metabolomics approach for clinical diagnosis of inborn errors of metabolism. Analytica Chimica Acta, 1025, 141–153. https://doi.org/10.1016/j.aca.2018.03.058

Kong, S. W., & Hernandez-Ferrer, C. (2019). Assessment of coverage for endogenous metabolites and exogenous chemical compounds using an untargeted metabolomics platform. Biocomputing, 587–598. https://doi.org/10.1142/9789811215636_0052

Liang, L., Ye, J., Han, L., Qiu, W., Zhang, H., Yu, Y., et al. (2020). Examining the blood amino acid status in pretherapeutic patients with hyperphenylalaninemia. Journal of Clinical Laboratory Analysis, 34(3), e23106. https://doi.org/10.1002/jcla.23106

Lumbreras, B., Porta, M., Márquez, S., Pollán, M., Parker, L. A., & Hernández-Aguado, I. (2008). QUADOMICS: An adaptation of the Quality Assessment of Diagnostic Accuracy Assessment (QUADAS) for the evaluation of the methodological quality of studies on the diagnostic accuracy of ’-omics’-based technologies. Clinical Biochemistry, 41(16–17), 1316–1325. https://doi.org/10.1016/j.clinbiochem.2008.06.018

Miller, M. J., Kennedy, A. D., Eckhart, A. D., Burrage, L. C., Wulff, J. E., Miller, L. A. D., et al. (2015). Untargeted metabolomic analysis for the clinical screening of inborn errors of metabolism. Journal of Inherited Metabolic Disease, 38(6), 1029–1039. https://doi.org/10.1007/s10545-015-9843-7

Moritz, L., Klotz, K., Grünert, S. C., Hannibal, L., & Spiekerkoetter, U. (2023). Metabolic phenotyping in phenylketonuria reveals disease clustering independently of metabolic control. Molecular Genetics and Metabolism, 138(3), 107509. https://doi.org/10.1016/j.ymgme.2023.107509

Mütze, U., Beblo, S., Kortz, L., Matthies, C., Koletzko, B., Bruegel, M., et al. (2012). Metabolomics of dietary fatty acid restriction in patients with phenylketonuria. PLoS ONE, 7(8), e43021. https://doi.org/10.1371/journal.pone.0043021

Pan, Z., Gu, H., Talaty, N., Chen, H., Shanaiah, N., Hainline, B. E., et al. (2007). Principal component analysis of urine metabolites detected by NMR and DESI-MS in patients with inborn errors of metabolism. Analytical and Bioanalytical Chemistry, 387(2), 539–549. https://doi.org/10.1007/s00216-006-0546-7

Schoen, M. S., & Singh, R. H. (2022). Plasma metabolomic profile changes in females with phenylketonuria following a camp intervention. Am J Clin Nutr, 115(3), 811–821. https://doi.org/10.1093/ajcn/nqab400

Schulpis, K. H., Karikas, G. A., Tjamouranis, J., Michelakakis, H., & Tsakiris, S. (2002). Acetylcholinesterase Activity and Biogenic Amines in Phenylketonuria. Clinical Chemistry, 48(10), 1794–1796. https://doi.org/10.1093/CLINCHEM/48.10.1794

Stroup, B. M., Nair, N., Murali, S. G., Broniowska, K., Rohr, F., Levy, H. L., & Ney, D. M. (2018). Metabolomic Markers of Essential Fatty Acids, Carnitine, and Cholesterol Metabolism in Adults and Adolescents with Phenylketonuria. The Journal of Nutrition, 148(2), 194–201. https://doi.org/10.1093/jn/nxx039

Václavík, J., Coene, K. L. M., Vrobel, I., Najdekr, L., Friedecký, D., Karlíková, R., et al. (2018). Structural elucidation of novel biomarkers of known metabolic disorders based on multistage fragmentation mass spectra. Journal of Inherited Metabolic Disease, 41(3), 407–414. https://doi.org/10.1007/s10545-017-0109-4

Wan, Z., Rosenbaum, E. R., Liu, W., Song, B., Yue, X., Kong, Y., et al. (2022). Benchmark Examination of Blood Amino Acids Patterns in Phenylketonuria Neonates and Young Children on Phenylalanine-Restricted Dietary Treatment. Fetal and Pediatric Pathology, 41(3), 443–450. https://doi.org/10.1080/15513815.2020.1846647

Weigel, C., Kiener, C., Meier, N., Schmid, P., Rauh, M., Rascher, W., & Knerr, I. (2008). Carnitine status in early-treated children, adolescents and young adults with phenylketonuria on low phenylalanine diets. Annals of Nutrition and Metabolism, 53(2), 91–95. https://doi.org/10.1159/000165356

Xiong, X., Sheng, X., Liu, D., Zeng, T., Peng, Y., & Wang, Y. (2015). A GC/MS-based metabolomic approach for reliable diagnosis of phenylketonuria. Analytical and Bioanalytical Chemistry, 407(29), 8825–8833. https://doi.org/10.1007/s00216-015-9041-3
